# Boosted CO_2_ Photoreduction Performance by CdSe Nanoplatelets via Se Vacancy Engineering

**DOI:** 10.1002/advs.202413684

**Published:** 2025-02-07

**Authors:** Huanhuan Luo, Xuanzhao Lu, Yue Cao, Zhaoyuan Lyu, Shichao Ding, Yuehe Lin, Yang Zhou, Wenlei Zhu, Yuanyuan Wang

**Affiliations:** ^1^ State Key Laboratory of Coordination Chemistry State Key Laboratory of Pollution Control and Resource Reuse State Key Laboratory of Analytical Chemistry for Life Science the Frontiers Science Center for Critical Earth Material Cycling School of Chemistry and Chemical Engineering School of the Environment Nanjing University Nanjing 210023 China; ^2^ School of Mechanical and Materials Engineering Washington State University Pullman WA 99164 USA; ^3^ State Key Laboratory for Organic Electronics & Information Displays Institute of Advanced Materials Nanjing University of Posts & Telecommunications Nanjing 210046 China

**Keywords:** CdSe NPLs, CO_2_ reduction, photocatalysis, Se vacancies, surface engineering

## Abstract

2D metal‐chalcogenide nanoplatelets (NPLs) exhibit promising photocatalysis properties due to their ultrathin morphology, high surface‐to‐volume ratio, and enhanced in‐plane electron transport mobility. However, NPLs, especially cadmium chalcogenides, encounter challenges in CO_2_ photoreduction due to insufficient solar energy utilization and fast recombination of photogenerated charge carriers. Defect engineering offers a potential solution but often encounters difficulties maintaining structural integrity, mechanical stability, and electrical conductivity. Herein, by taking two monolayers (2ML) CdSe NPLs as a model system, selenium (Se) vacancies confined in atomic layers can enhance charge separation and conductivity. A straightforward approach to create Se vacancies in various monolayers CdSe NPLs (2, 4, and 5ML) has been developed, enabling efficient CO_2_ photoreduction with a 4‐fold increase in CO generation compared to their defect‐free counterparts. Significantly, accounting for higher charge density and efficient carrier transport due to Se vacancies, defective 2ML CdSe NPLs (V_Se_‐2ML CdSe) exhibit CO evolution performance up to 2557.5 µmol g^−^¹ h^−^¹ with no significant decay over 5 h, which is an order of magnitude higher than that of common semiconductor catalysts. This study establishes a practical way to design advanced 2D semiconductor photocatalysts to achieve efficient CO_2_ photoreduction via defect engineering.

## Graphical abstract

A direct defect engineering approach is demonstrated to obtain CdSe nanoplatelets with Se vacancies. These nanoplatelets exhibit highly efficient photocatalytic CO₂ reduction performance, achieving a CO evolution rate of up to 2557.5 µmol g^−^¹ h^−^¹.

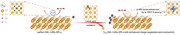

## 1 Introduction

Humanity's dependence on fossil fuels has led to significant CO_2_ emissions, resulting in global climate change and an energy crisis.^[^ ^1^ ^]^ Current methods to mitigate CO_2_ emissions include reduction, capture, storage, and conversion into value‐added chemicals.^[^ ^2^ ^]^ Among these, photocatalytic conversion of CO_2_ into hydrocarbon fuels under ambient conditions presents a promising approach, which reduces environmental pollution and simultaneously solves the energy crisis.^[^ ^3^, ^4^, ^5^ ^]^ To date, various semiconductors, such as perovskite,^[^ ^6^ ^]^ TiO_2_,^[^ ^7^ ^]^ Bi_2_O_2_CO_3_,^[^ ^8^ ^]^ ZnIn_2_S_4_,^[^ ^9^ ^]^ and CdS^[^ ^10^ ^]^ have been utilized as photocatalysts in CO_2_ reduction. However, these semiconductors suffer from insufficient photoconversion efficiency in practical applications. The critical step of photoconverting CO_2_ is separating and transferring charge carriers within the photocatalysts.^[^ ^11^ ^]^ Therefore, it is essential to improve electron‐hole separation and decrease the recombination rate of photogenerated charge carriers to enhance the efficiency of solar‐driven CO_2_ conversion.

In bulk materials, charge separation is more challenging to achieve than charge recombination, as the timescale for charge recombination typically occurs within a few picoseconds (ps), whereas charge separation generally requires a much longer timescale, often several hundred picoseconds.^[^ ^12^ ^]^ Therefore, developing atomic‐level strategies to enhance charge separation is critical for improving catalytic performance and reducing recombination losses. Reducing the charge diffusion distance was one efficient strategy,^[^ ^13^ ^]^ while 2D metal‐chalcogenide nanocrystals (nanoplatelets, NPLs), the atomically thin material^[^ ^14^, ^15^ ^]^ exhibited outstanding ability in facilitating charge separation^[^ ^16^, ^17^, ^18^, ^19^ ^]^ and enriching reactive sites.^[^ ^16^, ^20^, ^21^, ^22^ ^]^ However, research on CO_2_ photocatalysis using semiconductor NPLs is still in its infancy. Significant challenges remain in developing efficient CO_2_ photocatalysts based on NPLs due to insufficient solar energy utilization, fast recombination of photoinduced electron‐hole pairs, and inefficient adsorption and photoconversion of CO_2_^[^ ^23^ ^]^.

Though 2D materials coupled with a defect‐free surface could be suitable for electron transport, CO_2_ adsorption, and photoconversion prefer defect surfaces.^[^ ^24^ ^]^ Therefore, developing 2D materials coupled with defect surfaces was expected to be a promising strategy to convert CO_2_ in high activity. Indeed, defect engineering has been demonstrated to be an efficient strategy for tuning the CO_2_ photoconversion efficiency of semiconductors, including metal oxides, metal chalcogenides, etc.^[^ ^25^ ^]^ The defects could act as centers for trapping photoexcited electrons, thereby restricting the photocarrier recombination. In addition, the electron structure of the semiconductor could be adjusted to tune the binding energy of active intermediates, which is beneficial for CO_2_ photoconversion. For example, Xie et al. introduced Zn vacancies to ZnIn_2_S_4_ NPLs by adjusting the temperature of its synthesis process, thereby boosting the performance of CO_2_ photoconversion by three times.^[^ ^26^ ^]^ Therefore, it is theoretically possible to design CdSe NPLs with appropriate defects for efficient CO_2_ photoreduction by utilizing the structural advantages and defect engineering. However, constructing defects within NPLs while maintaining their structural integrity, mechanical stability, and electrical conductivity is challenging.^[^ ^27^, ^28^ ^]^ Besides, controlling the defect density toward customized defect distribution for specific applications was demanding, significantly maximizing its density. Traditional direct synthesis methods such as chemical vapor transport (CVT) and chemical vapor deposition (CVD) can lead to an in‐plane defect density gradient or phase separation in the materials,^[^ ^29^ ^]^ while post‐growth defect treatment methods regarding spatial control over material functionality and phase maintenance are limited.^[^ ^30^ ^]^ Thus, exploring a milder and more controllable method to fabricate designed defect‐engineered CdSe NPLs is essential.

Bearing these in mind, we focused on designing deficient 2D CdSe NPLs to achieve efficient CO_2_ photoconversion. We developed a gentle and controllable chemical oxidation strategy for creating Se vacancies in CdSe NPLs to boost its activation of CO_2_ photoreduction. The oxidation and Lewis acidity of NO^+^ ions could be utilized to remove surface ligands and simultaneously introduce selenium (Se) vacancies into CdSe NPLs (V_Se_‐CdSe NPLs) while maintaining morphology and enhancing electricity conductivity. The treated 2ML CdSe NPLs exhibited overwhelming photocatalytic performance on gas‐solid CO_2_ reduction. The CO evolution rate as high as 2557.5 µmol g^−1^ h^−1^ could be achieved without any sacrificial reagents, which has been a record for CO_2_ photoreduction to CO under visible light. Further studies revealed that after introducing Se vacancies, defective 2ML CdSe NPLs exhibit enhanced performance in light absorption, charge transfer, and charge separation. The total density of state (DOS) calculation indicated that more electrons were near the Fermi level in V_Se_‐2ML CdSe NPLs than its defectless counterpart, suggesting a promising stronger interaction between its surface and CO_2_/H_2_O, facilitating easy electron transfer. In addition, in situ Fourier‐transform infrared (FTIR) spectroscopy further confirmed this and demonstrated that the formation of CO was achieved through a two‐electron reduction pathway by capturing COOH^*^ intermediate. Furthermore, we observed that defective CdSe NPLs (2, and 5ML CdSe NPLs) could significantly enhance the performance of CO_2_ photoreduction, achieving four times the efficiency of pristine NPLs. Thus, we proved that the atomically thin CdSe NPLs with proper Se vacancies are efficient for CO_2_ photoconversion. Besides, we developed a simple and versatile strategy that effectively introduced defects into NPLs, providing a new strategy for designing novel defective structural 2D semiconductor photocatalysts that pursue high CO_2_ photoconversion efficiency.

## 2 Theoretical Study

**Figure 1:**
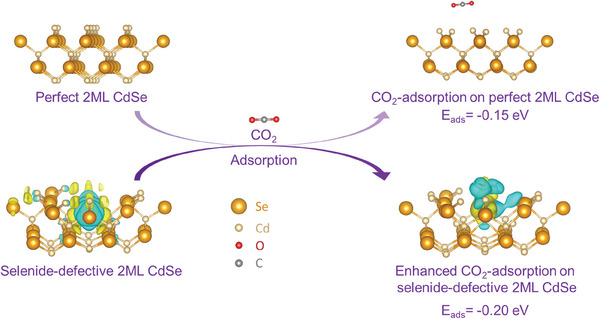
Theoretical study on charge distribution and separation in 2ML CdSe nanoplatelets (NPLs) with Se vacancies (V_Se_). Schematic illustration of the adsorption of CO_2_ molecules onto perfect 2MLCdSe NPLs and Se‐defective V_Se_‐2ML CdSe NPLs with the partial charge density of Se vacancies. Electrons neighboring Se vacancies are more localized in V_Se_‐2ML CdSe. The yellow and blue isosurfaces represent charge accumulation and depletion in the space. Methods in support information (SI) provided more information on the calculation. Color legends: light yellow, Cd; golden, Se; rose red, O; gray, C.

Herein, considering the 2ML CdSe NPLs with Se vacancies (V_Se_) as an example, we studied the role of Se deficiency in charge separation and distribution in 2ML CdSe NPLs. The electronic structures were investigated via density functional theory (DFT) calculations. According to the density of states (DOS), the existence of V_Se_ resulted in the *d*‐band centers being closer to the Fermi Level, for which the *d*‐band centers were calculated to be −8.43 eV for 2ML CdSe and −8.245 eV for V_Se_‐2ML CdSe (Figure S3a,b, Supporting Information). This indicated that more electrons were around the Fermi level of V_Se_‐2ML CdSe and suggested a promising stronger interaction between its surface and CO_2_/H_2_O, enabling easy electron transfer. In addition, the differences of charge density between 2ML CdSe and its defective counterpart V_Se_‐2ML CdSe (left part of **Figure** **1**) were theoretically determined, which indicated that the electrons neighboring Se vacancies could be localized (Figure 1), suggesting electrons in the defect structure, V_Se_‐2ML CdSe, are more likely to be excited. Readily excited electrons in the conduction band would benefit the photocatalyst by improving its activation ability toward CO_2_ and H_2_O molecules and boosting its conductivity, ultimately facilitating CO_2_ reduction (CO_2_RR).^[^ ^31^ ^]^ In the presence of Se vacancies, we indeed found a much stronger interaction between CO_2_ and V_Se_‐2ML CdSe, for which the adsorption energy of CO_2_ showed enhancement from −0.15 eV (2ML CdSe NPLs) to −0.20 eV (V_Se_‐2ML CdSe NPLs) after the introducing of Se vacancies (right part of Figure 1), which would facilitate the following CO_2_ reduction processes. Based on our findings, it is reasonable to hypothesize that 2ML CdSe NPLs with Se vacancies would be efficient catalysts to improve CO_2_ photoconversion efficiency (Figure S3c, Supporting Information).

## 3 Synthesis and Characterization of Defective CdSe NPLs

**Figure 2:**
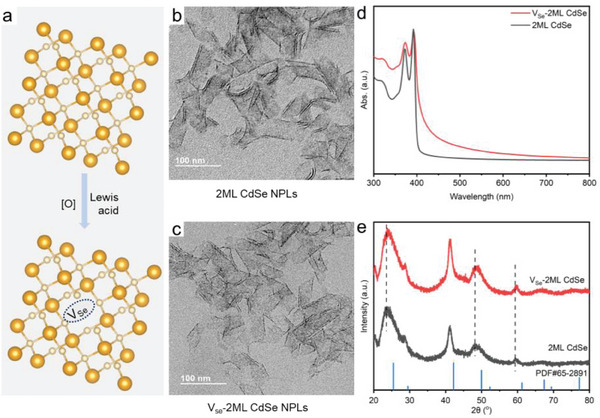
Synthesis and characterization of 2ML CdSe NPLs with Se vacancies. a) Schematic introduction of the preparation of 2ML CdSe NPLs with Se vacancies (V_Se_‐2ML CdSe NPL) utilizing the synergistic effect of oxidation and Lewis acidity through post‐treatment on 2ML CdSe NPLs. TEM images investigate morphology maintenance of as‐prepared b) 2ML CdSe NPLs and c) V_Se_‐2ML CdSe NPLs with Se vacancies, respectively. d) UV–vis absorption spectra to investigate the optical properties of 2ML CdSe NPLs and V_Se_‐2ML CdSe NPLs. e) XRD patterns of 2ML CdSe NPLs and V_Se_‐2ML CdSe NPLs. The scale bars in (b, c) are 100 nm. Color legends: light yellow, Cd; golden, Se.

In this work, colloidal 2ML CdSe NPLs with three layers of Cd, and two layers of Se were synthesized according to the hot injection method,^[^ ^26^ ^]^ while V_Se_‐2ML CdSe NPLs were obtained through post‐treated with NOBF_4_ having both oxidation and Lewis acidity properties (**Figure** **2**a). Details of sample preparation can be found in the experimental section and Supporting Information (SI).

The optical properties, microstructure, and morphology of V_Se_‐2ML CdSe were characterized by UV–vis absorption, X‐ray diffraction (XRD), and transmission electron microscope (TEM) (Figure 2). As shown in Figure 2d, V_Se_‐2ML CdSe possessed almost the same maximum absorption wavelength as 2ML CdSe,^[^ ^32^ ^]^ indicating that the composition and structure of the NPLs have mainly remained preserved. The absorption edge redshift observed in V_Se_‐2ML CdSe originating from the relaxation of surface strain stress, likely caused by ligand environment changing and defect creation, indicating promising enhanced photoresponse.^[^ ^32^, ^33^, ^34^, ^35^ ^]^ TEM images further confirmed the structural integrity of NPLs after treatment (Figure 2b,c). The XRD patterns of V_Se_‐2ML CdSe samples (Figure 2e) showed characteristic peaks at ≈23.7, 28.6, 41.2, and 48.2°, corresponding to the (111), (200), (220), and (311) planes of zincblende structures.^[^ ^36^ ^]^ These peaks are consistent with those of the pristine NPLs, indicating that the crystal structure remains unchanged during oxidation.

The Fourier transform infrared (FT–IR) and thermogravimetric analysis (TGA) were conducted to investigate changes in the ligand environment. After treatment with NOBF_4_, there was a significant decrease in characteristic peaks of COOH (≈1500 cm^−1^) and C─H stretching vibration (≈2900 cm^−1^) in V_Se_‐2ML CdSe (Figure S4, Supporting Information), as evidenced by TGA analysis, which showed a 20% reduction in organic ligand mass ratio (Figure S5, Supporting Information).^[^ ^37^ ^]^ As a stripping ligand agent, NOBF_4_ could effectively remove organic molecules from the surface of nanocrystals, thereby enhancing charge transfer efficiency.^[^ ^38^ ^]^ However, unlike the complete ligand stripping in 0D QDs, the 2ML CdSe NPLs treated with NOBF_4_ still partially retained organic ligands. This retention might be due to the rectangular NPLs' helical structure, which shields certain crystal facets and protects the ligands from being stripped away.^[^ ^39^, ^40^ ^]^ This partial ligand stripping effect allowed the CdSe NPLs to maintain their original morphology while enhancing electron transport efficiency.

**Figure 3:**
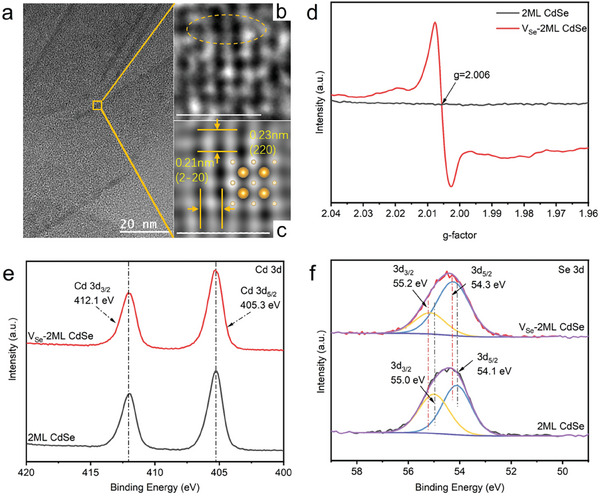
Structure characterizations for the defected controlled 2ML CdSe NPLs (2ML CdSe NPLs and V_Se_‐2ML CdSe NPLs). a, b) HRTEM images of V_Se_‐2ML CdSe, the orange circle in (b) highlights the lattice disorder. c) FFT pattern and the atomic scale of the crystalline phase of V_Se_‐2ML CdSe. d) Room temperature electron paramagnetic resonance (EPR) signals to investigate Se vacancies creation in V_Se_‐2ML CdSe after post‐treatment. High‐resolution X‐ray photoelectron spectroscopy (XPS) to illustrate surface chemical composition and elements' chemical value states of e) Cd 3d and f) Se 3d in 2ML CdSe, V_Se_‐2ML CdSe NPLs, respectively. Scale bars in (a–c) are 20, 1, and 1 nm, respectively. Color legends: light yellow, Cd; golden, Se.

To gain insight into the deficiency (V_Se_) of as‐prepared nanoplatelets, high‐resolution transmission electron microscopy (HRTEM) was carried out to show its fine structure. As shown in **Figure** **3**a–c, the nanoplatelets showed the interplanar spacings of 0.23  and 0.21 nm, corresponding well to the d_220_ and d_2‐20_ spacings, while the corresponding dihedral angle is ≈90°, aligning well with the calculated angle between the (220) and (2–20) planes of zincblende CdSe,^[^ ^41^ ^]^ consistent with the XRD characterization shown in Figure 2e, further confirming the crystal phase's consistency before and after surface treatment. Surprisingly, the HRTEM images (Figure 3b) show slight lattice disorder and atom dislocation, highlighted by yellow circles in the figure. We attributed this phenomenon to the presence of vacancies in V_Se_‐2ML CdSe NPLs, which aligned with previous reports on identifying P vacancies in Ni_0.96_Co_0.04_P^[^ ^42a^ ^]^ and vacancies in WO_3_.^[^ ^42b^ ^]^ At the same time, we observed the redshift of the Raman signal of Cd‐Se (LO, 204 cm^−1^) in V_Se_‐2ML CdSe NPLs compared to 2ML CdSe NPL (LO, 202 cm^−1^) (Figure S6, Supporting Information), which was related to the change of Cd/Se stoichiometric ratio.^[^ ^42c^ ^]^ As illustrated by ICP analysis, a larger Cd/Se ratio was observed in V_Se_‐2ML CdSe (Cd: Se = 1.55: 1) compared to 2ML CdSe (Cd: Se = 1.50: 1) (Table S1, Supporting Information). We inferred that Se vacancies should be created in 2ML CdSe after NOBF_4_ treatment. It was further proofed by electron paramagnetic resonance (EPR) analysis, which showed that only V_Se_‐2ML CdSe exhibited an EPR signal with g = 2.006 (Figure 3d).^[^ ^31^, ^43^ ^]^ In essence, the EPR signal (g = 2.006) originated from electrons trapped by Se vacancies, unambiguously demonstrating that NOBF_4_ could induce Se vacancies in 2ML CdSe NPLs due to its simultaneous oxidation property and Lewis acidity.

Surface chemical composition and the chemical value states of NPLs of elements were analyzed using X‐ray photoelectron spectroscopy (XPS) characterization. The binding energies of Cd 3d were almost the same for 2ML CdSe and V_Se_‐2ML CdSe (Figure 3e), with peaks corresponding to Cd^2+^ centered at 412.1  and 405.3 eV for Cd 3d_3/2_ and Cd 3d_5/2_, respectively. The high‐resolution XPS spectrum of Se 3d revealed two peaks at 55.0  and 54.1 eV for 2ML CdSe, assigned to Se 3d_3/2_ and Se 3d_5/2_ of Se^2−^. In V_Se_‐2ML CdSe, Se 3d binding energies blue‐shifted to 55.2 and 54.3 eV (Figure 3f), indicating the higher binding energy of Se 3d.^[^ ^44^ ^]^ Furthermore, the peak convolutional ratio of Se 3d_3/2_ in V_Se_‐2ML CdSe was lower than that of the 2ML CdSe NPLs. According to existing literature,^[^ ^45a,b^ ^]^ in some cases, the introduction of anion vacancies (such as Se vacancies) may cause minimal impact on the cationic species, which means it is possible to detect no significant shift in the binding energy of Cd 3d binding energy. Therefore, the blue shift in Se 3d binding energy and decrease in the peak convolutional ratio of Se 3d_3/2_ were ascribed to the Se vacancy in the V_Se_‐2ML CdSe.^[^ ^45^, ^46^ ^]^ Additionally, the blueshift of Se 3d binding energy indicated that the fermi level movement toward the conduction band direction, akin to n‐type doping, facilitates electron transport.^[^ ^34^, ^47^ ^]^ Hence, the above results have proved that V_Se_‐2ML CdSe NPLs with Se vacancies have been successfully designed assisting by NOBF_4_.

This strategy could be extended to other agents armed with simultaneous oxidation properties and Lewis acidity, such as In(NO_3_)_3_ or plasma treatment under air ([air]‐plasma). The Se vacancies on CdSe NPLs could also be created by In(NO_3_)_3_ and [air]‐plasma effectively while maintaining its 2D NPLs morphology and crystal structure. As evidenced by UV–vis‐DRS (Figure S7, Supporting Information), TEM images (Figure S8, Supporting Information), and XRD characterization (Figure S9, Supporting Information), the structural integrity and crystal structure of NPLs kept well after these two treatments. Raman signal at 204 cm^−1^ (LO) (Figure S10, Supporting Information) was also observed in 2ML CdSe‐In(NO_3_)_3_ and 2ML CdSe‐plasma samples, respectively, which was the same as V_Se_‐2ML CdSe, illustrating the change of Cd: Se ratio. Besides, a slightly higher Cd/Se ratio (Cd: Se = 1.56: 1) was detected in 2ML CdSe‐In(NO_3_)_3_, which not only confirmed the Cd/Se ratio change but also helped to infer that Se vacancies should be created in 2ML CdSe after treated with In(NO_3_)_3_. Fortunately, Se vacancies in 2ML CdSe‐In(NO_3_)_3_ and 2ML CdSe‐plasma were pointed out directly by EPR testing and XPS element analysis. EPR signals of 2ML CdSe‐In(NO_3_)_3_ and 2ML CdSe‐plasma were similar to V_Se_‐2ML CdSe with g = 2.006 (Figure S11, Supporting Information), and high‐resolution XPS Se 3d (Figure S12a, Supporting Information) and Cd 3d (Figure S12b, Supporting Information) for both were also similar to V_Se_‐2ML CdSe. These demonstrated the efficiency and universality of introducing Se vacancies through Lewis acidity and oxidization. Furthermore, this strategy was also effective for other NPLs, such as 5ML CdSe NPLs, for which there was not an obvious change of 2D NPLs morphology and composition for 5ML CdSe‐In(NO_3_)_3_ and 5ML CdSe‐NOBF_4_, as evidenced UV–vis absorption spectra and TEM images (Figures S13, S14, Supporting Information). Besides, the EPR signal at g = 2.006 of V_Se_‐5ML CdSe (Figure S15a, Supporting Information) proved Se vacancies existed, which would be further demonstrated by HRTEM images of 5ML CdSe‐In(NO_3_)_3_ (Figure S16, Supporting Information). Therefore, it should be reasonable that Se vacancies could be introduced through Lewis acidity and oxidation.

## 4 Photocatalytic Performances in CO_2_ Reduction and Mechanistic Investigation

**Figure 4:**
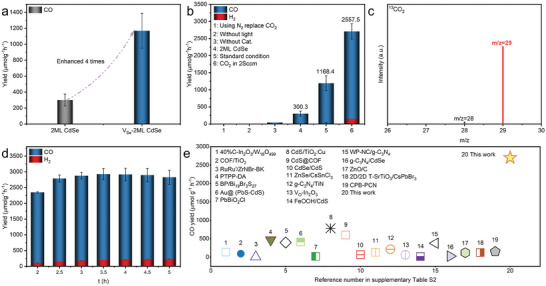
Photocatalytic performance study. Photoreduction of CO_2_ into CO in the online system a) under 300 W Xe lamp irradiation for the 2ML CdSe and V_Se_‐2ML CdSe. b) Control experiments of photoreduction of CO_2_ into CO. c) Mass spectrum of ^13^CO generated from the photoreduction of the ^13^CO_2_ isotopic experiment. d) Stability of the CO production rates on V_Se_‐2ML CdSe under continuous light irradiation. e) Comparison of the efficiency of recently published references on photoreduction CO_2_ in gas‐solid systems. Detailed information is shown in Table S2 (Supporting Information).

As mentioned above, V_Se_‐2ML CdSe with Se vacancies processed enhanced photo absorbance ability and efficient electron transport, which should be promising for efficient CO_2_ photoreduction. Herein, the photocatalytic CO_2_ reduction performance of as‐prepared CdSe NPLs was tested on an online photocatalytic test system in a gas‐solid setup without any sacrificial agents under light irradiation (Scheme S1, Supporting Information). CO was the major product, with minor CH_4_ and H_2_ byproducts, owing to the inherent kinetic advantage of the two‐electron reduction pathway over the multiple‐electron reduction process.^[^ ^48^ ^]^ In other words, the formation of CH_4_ via eight electron reduction and water splitting was highly unlikely to occur in the gas‐solid reaction setup.^[^ ^49^ ^]^ CO evolution rates were 1168.4and 300.3 µmol g^−1^ h^−1^ for V_Se_‐2ML CdSe and 2ML CdSe, respectively (**Figure** **4**a). The performance increased ≈4 times through the introduction of defects (Se‐vacancies) into 2ML CdSe. Besides, the absorbance edge of V_Se_‐2ML CdSe would red‐shift gradually with the increasing concentration of NOBF_4_ (Figure S17, Supporting Information), suggesting that the concentration of defects could be tuned by adjusting the concentration of NOBF_4_. The CO_2_ photoconversion efficiency of the V_Se_‐2ML CdSe‐x (x means 2ML CdSe treated with NOBF_4_ in different concentrations) showed a volcano‐like trend relative to the concentration of NOBF_4_ (Figure S18, Supporting Information), with V_Se_‐2ML CdSe‐40 displayed the highest CO evolution rate. This observation aligned with our theoretical calculations, which also showed that the d‐band center energy level of the V_Se_‐2ML CdSe exhibited a volcano‐shaped distribution as vacancy concentration increased (Figure S3b, Supporting Information). These results indicated that photocatalysts with preferable CO_2_ photoreduction performance could be easily obtained by changing the concentration of ligand stripping agents according to changing the concentration of Se vacancies, such as NOBF_4_.

Control experiments showed no detectable products with purging N_2_ or without light or V_Se_‐2ML CdSe (Figure 4b). This illustrated that the CO_2_ photoreduction relies on light‐induced photocatalyst (V_Se_‐2ML CdSe), with CO_2_ as a carbon source. It was further confirmed by the ^13^CO_2_ labeling experiment, in which the dominant product was ^13^CO (m/z = 29) during the purging of pure ^13^CO_2_ (99.0%) gas (Figure 4c). Furthermore, we found that varying CO_2_ flow rates significantly impacted CO_2_ photoreduction, with two times increase in CO evolution rates by reducing flow rate from 8.0to 2.0 Sccm (Figure 4b and Figure S19, Supporting Information), which indicated that a lower flow rate enhances CO_2_/H_2_O molecule adsorption onto the photocatalyst surface, affecting the gas reactants mole ratio^[^ ^8^, ^26^, ^50^ ^]^ and mass transfer,^[^ ^51^ ^]^ thus influencing CO_2_ photoreduction efficiency. Besides, apparent quantum yields (AQY) were measured under different monochromatic light wavelengths to evaluate the photocatalytic activity of CO evolution. As shown in Figure S20 (Supporting Information), the AQY response of V_Se_‐2ML CdSe matches well to its UV–vis spectrum, achieving an AQY of 1.32% at 400 nm. Interestingly, as shown in Figure S21 (Supporting Information), the sample, V_Se_‐2ML CdSe (5.9 m^2^ g^−1^) processed a slightly larger BET surface area than 2ML CdSe (0.9 m^2^ g^−1^), which not only indicated that V_Se_‐2ML could expose more surface to participate in the reactivity but also indicated that the enhanced performance on CO_2_ photoreduction could also be attributed to the induced Se vacancies.

The stability of production of CO initiated by V_Se_‐2ML CdSe was tested over 5 h, showing no significant decrease (Figure 4d). The CO photocatalytic evolution performance of the V_Se_‐2ML CdSe sample (2557.5 µmol g^−1^ h^−1^) without cocatalysts or sacrificial agents was the best one among reports in the solid‐gas system (Figure 4e and Table S2, Supporting Information). Our findings once again confirmed that defects (Se vacancies) significantly enhance its CO_2_ photoreduction activity. In addition, the photocatalytic CO_2_ reduction was tested for four consecutive runs (Figure S22, Supporting Information) to further demonstrate the stability of V_Se_‐2ML CdSe. After each cycle, the light irradiation was removed with no catalyst washing, while keeping CO_2_ flow. There was nearly no significant loss in photocatalytic activity after four cycles, which demonstrated its photostability again.

To further elucidate the photostability of V_Se_‐2ML CdSe, its optical properties, chemical composition, and morphology were conducted after photocatalysis. XRD characterization (Figure S23, Supporting Information) and TEM images (Figure S24, Supporting Information) showed maintained structure and morphology of V_Se_‐2ML CdSe after photocatalysis (V_Se_‐2ML CdSe‐Cat), supported by UV–vis spectra (Figure S25, Supporting Information). Its chemical composition was also kept well according to Raman (Figure S26, Supporting Information) and high‐resolution XPS Cd 3d and Se 3d analysis (Figure S27, Supporting Information). TGA (Figure S28, Supporting Information) and FTIR (Figure S29, Supporting Information) analyses suggested that the organic ligand of V_Se_‐2ML CdSe didn't decompose, and CO_2_ was the only carbon source. Besides, there was no obvious discrepancy in the EPR signal (Figure 3d and Figure S30, Supporting Information) or high‐resolution XPS characterization (Figure S27, Supporting Information). V_Se_‐2ML CdSe emerges as a promising photocatalyst for CO_2_ photoreduction based on its demonstrated photostability and high activity.

**Figure 5:**
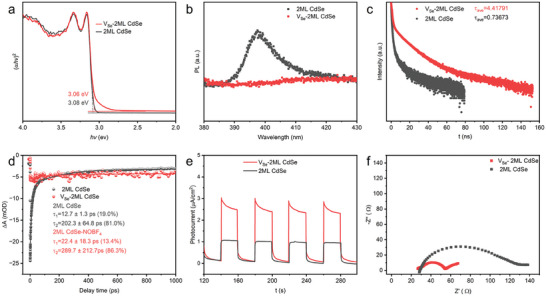
Advantages of Se vacancies confined in 2ML CdSe layers. a) Tauc plot to illustrate the bandgap narrowing in V_Se_‐2ML CdSe NPLs. b) Photoluminescent (PL) intensity, c) time‐resolved PL decay plots (TRPL), and d) ultrafast transient absorption (TA) spectra to elaborate the enhanced photogenerated carrier separation in V_Se_‐2ML CdSe NPLs. e) Transient photocurrent (PC) and f) electrochemical impedance spectra (EIS) to illustrate the enhanced separation and transportation of photogenerated carriers in V_Se_‐2ML CdSe.

The separation and transportation behaviors of photoinduced charges were surveyed to understand the origin of significantly promoted CO_2_ photoreduction activity by induced Se vacancies. Band structure variety of 2ML CdSe after introducing Se vacancies was explored, which was commonly affected by defect engineering and played a key role in the CO_2_ photoreduction performance. The UV–vis absorption spectra (Figure 2d) revealed a redshift in the absorption edge of V_Se_‐2ML CdSe compared to 2ML CdSe, expanding the photoabsorbance to the visible‐light region, and facilitating its photoresponse. It also indicated that defects (Se vacancies) in V_Se_‐2ML CdSe could introduce midgap states into its band structure, confirmed by UV–vis‐DRS characterization. It was obvious that the bandgap of 2ML CdSe (*Eg* = 3.08 eV) was slightly larger than that of V_Se_‐2ML CdSe (*Eg* = 3.06 eV) (**Figure** **5**a), attributed to the existence of defects (Se vacancy) in V_Se_‐2ML CdSe. XPS valence band spectra were carried out to determine the VB maximum position with respect to their *E_F_* for V_Se_‐2ML CdSe and 2ML CdSe, which were 0.85 and 0.86 eV. Considering the optical band gaps, we could obtain the electronic band energies relative to a normal hydrogen electrode (Figure S31, Supporting Information), indicating that both the V_Se_‐2ML CdSe and 2ML CdSe possessed the ability to realize CO_2_ photoreduction and O_2_ evolution simultaneously, which has been illustrated through CO_2_ photoreduction experiment (Figure 4a), while O_2_ was indirect test by capturing the hydroxy radical (·OH) (Detailed in part of Figure S32, Supporting Information).

The photoluminescence intensity (PL) and time‐resolved PL decay (TRPL) were conducted to elaborate the photogenerated carrier separation, as photoinduced electron and hole recombination emits fluorescence. Lower PL emission intensity at 397 nm in V_Se_‐2ML CdSe compared to 2ML CdSe (**Figure** **5**b) could suggest efficient photogenerated carrier separation in V_Se_‐2ML CdSe, coinciding with the prior report.^[^ ^52^ ^]^ This indicated enhanced CO_2_ photoreduction in V_Se_‐2ML CdSe could be attributed to the suppression of photocarrier recombination.^[^ ^53^ ^]^ The transport dynamics of photogenerated carriers were revealed by a time‐resolved PL decay (TRPL) plot (Figure 5c). PL lifetime increased from 0.74 ns (2ML CdSe) to 4.42 ns (V_Se_‐2ML CdSe), indicating that photogenerated electrons of V_Se_‐2ML CdSe on the bottom of the conduction band (CB) could be trapped by defects (Se vacancies) and then undergo trap‐to‐trap hopping rather than radiation recombination. It was further confirmed that defects (Se vacancies) in V_Se_‐2ML CdSe were useful in restricting the recombination of photogenerated e^−^ and h^+^.^[^ ^48^, ^54^ ^]^ Ultrafast transient absorption spectroscopy (TA) was also applied to unveil the photocarriers' separation, transport, and recombination.^[^ ^55^, ^56^ ^]^ Both 2ML CdSe and V_Se_‐2ML CdSe exhibited stimulated emission (SE) signal (Figure 5d), with longer weighted averaged lifetime and slower decay component τ_2_ in V_Se_‐2ML CdSe compared with 2ML CdSe. The enhanced performance in CO_2_ photoreduction of V_Se_‐2ML CdSe could be attributed to Se vacancies acting as trap centers for photoexcited electrons, improving the separation efficiency of photogenerated electron‐hole pairs and increasing electron participation in CO_2_ photoconversion.

The separation and transportation behaviors of the photocarrier were further explored through transient photocurrent (PC) analysis^[^ ^57^ ^]^ and electrochemical impedance spectra (EIS) characterization.^[^ ^58^ ^]^ As shown in Figure 5e, the photocurrent density of V_Se_‐2ML CdSe with defects was higher than that of 2ML CdSe under light irradiation. Additionally, the arc radius of Nyquist plots of V_Se_‐2ML CdSe was smaller than that of 2ML CdSe (Figure 5f). These characterizations revealed reduced recombination of photoinduced electrons and holes. The promoted separation efficiency, which was coincident with the characterization of UV–vis‐DRS (Figure 5a), PL (Figure 5b), TRPL (Figure 5c), and TA (Figure 5d). Therefore, modification of 2ML CdSe NPLs with defects effectively reduced the recombination of photoinduced charge carriers, promoting their separation efficiency and transfer, beneficial for CO_2_ photoreduction.

## 5 Universality of Se Vacancy in CdSe NPLs

**Figure 6:**
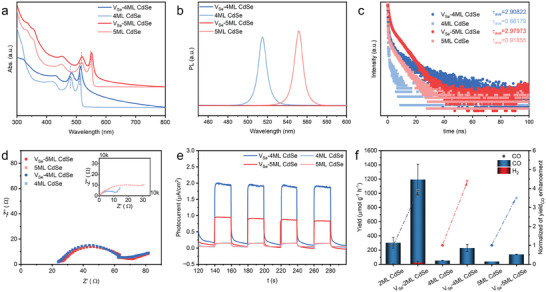
Extension of the Se vacancies creation in other NPLs. a) UV–vis spectra to investigate the broad photoresponse in V_Se_‐CdSe NPLs (V_Se_‐4ML CdSe, V_Se_‐5ML CdSe). b) PL intensity and c) time‐resolved PL decay plots (TRPL) to elaborate the enhanced photogenerated carrier separation in V_Se_‐CdSe NPLs (V_Se_‐4ML CdSe, V_Se_‐5ML CdSe). d) Electrochemical impedance (EIS) spectra and e) Transient photocurrent (PC) spectra to illustrate the enhanced separation and transportation of photogenerated carrier in V_Se_‐CdSe NPLs (V_Se_‐4ML CdSe, V_Se_‐5ML CdSe). f) Photoreduction of CO_2_ into CO in an online system over different CdSe NPLs.

We have demonstrated that introducing defects (Se vacancies) in 2ML CdSe NPLs can enhance charge separation efficiency, accelerate charge transfer, and extend the lifetime of photogenerated carriers, resulting in superior CO_2_ photoreduction performance. To verify the generality of this approach, we treated 4ML CdSe and 5ML CdSe samples with NOBF_4_ and EPR testing (Figure S15, Supporting Information) have demonstrated that NOBF_4_ could introduce Se vacancies in these two NPLs, which was further proved by ICP testing (Table S1, Supporting Information) for a little increase of Cd/Se mole ratio in V_Se_‐4ML CdSe NPLs (Cd: Se = 1.27: 1) samples comparing to 4ML CdSe NPLs (Cd: Se = 1.25: 1). Besides, we studied the effects of Se vacancies on their optical properties, charge carrier dynamics, and photoresponse. First, the absorbance edges and maximum absorption wavelength were red‐shifted for V_Se_‐4ML CdSe and V_Se_‐5ML CdSe, indicating the enhanced photoresponse ability. Besides, the 2D nanocrystal morphology retained well according to UV–vis absorption (**Figure** **6**a) and TEM images (Figures S13, S33, Supporting Information). Second, the PL intensity of V_Se_‐4ML CdSe and V_Se_‐5ML CdSe decreased dramatically compared to 4ML CdSe and 5ML CdSe (Figure 6b), respectively. Furthermore, introducing defects also led to an increase in the average lifetime (Figure 6c) for V_Se_‐4ML CdSe and V_Se_‐5ML CdSe. It was indicated that the recombination of photoinduced charge carriers was restricted in V_Se_‐4ML CdSe and V_Se_‐5ML CdSe. Third, EIS and PC characterizations proved that the defects in CdSe NPLs enhanced photogenerated charge separation efficiency and charge transfer by introducing a mid‐gap state into the band structure. As shown in Figure 6d,e, the arc diameter of the Nyquist plot was smaller, and the photocurrent was higher for V_Se_‐4ML CdSe and V_Se_‐5ML CdSe compared to their untreated counterparts.

The performance of CO_2_ photoconversion for defective CdSe NPLs with different thicknesses was investigated to evaluate the universality of Se‐vacancy‐related enhancement of CO_2_ photoreduction efficiency. Although the CO_2_ photoreduction performance of thicker NPLs was lower than V_Se_‐2ML CdSe, the trend in the increase of CO photocatalytic evolution rates after defect introduction was similar: V_Se_‐4ML CdSe (229.0 µmol g^−1^ h^−1^) > 4ML CdSe (52.5 µmol g^−1^ h^−1^), V_Se_‐5ML CdSe (138.0 µmol g^−1^ h^−1^) > 5ML CdSe (40.0 µmol g^−1^ h^−1^). All V_Se_‐CdSe NPLs exhibited a ≈4‐fold increase in CO production rate after NOBF_4_ treatment, as evident from the normalized data (Figure 6f).

## 6 Pathway of CO_2_ Reduction

**Figure 7:**
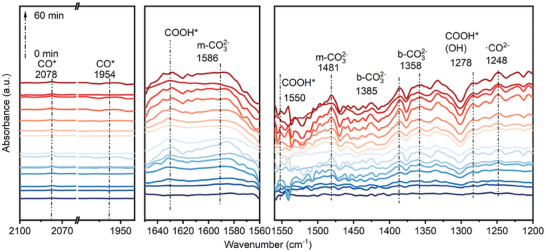
Illustration of CO_2_ photoreduction into CO over V_Se_‐2ML CdSe. In situ DRIFTS of V_Se_‐2ML CdSe in the presence of CO_2_ and H_2_O vapor within 60 min illumination.

To elucidate the CO_2_ reduction pathway, the reaction intermediates were captured through an in situ Fourier‐transform infrared (in situ FT–IR) experiment (**Figure** **7**; Figure S34, Supporting Information). The signal intensity in the 1000–2000 cm^−1^ region increased drastically along with the light irradiation for both samples (Figure 7; Figure S34, Supporting Information). ″In the V_Se_‐2ML CdSe sample, the peaks at 1358 and 1385 cm^−^¹, and in the 2ML CdSe sample, the peaks at 1355 and 1389 cm^−^¹, could be attributed to the formation of bidentate bicarbonate (b‐CO₃^2^^−^) as a result of the reaction between CO₂ and H₂O. Meanwhile, the peaks observed at 1481 and 1586 cm^−^¹ in the V_Se_‐2ML CdSe sample, and at 1484 and 1586 cm^−^¹ in the 2ML CdSe sample were characteristic of monodentate bicarbonate (m‐CO₃^2^^−^) species.^[^ ^59^ ^]^ In addition, the peaks at 1248 cm^−^¹ in V_Se_‐2ML CdSe and 1246 cm^−^¹ in 2ML CdSe aligned well with the vibrational frequencies of carboxylate groups (CO_3_^2−^),^[^ ^59a^ ^]^ indicating that both samples follow a similar reaction pathway. However, distinct peaks at 1278 and 1550 cm^−^¹ were observed in the V_Se_‐2ML CdSe sample (Figure 7), which could be assigned to the COOH group^*^.^[^ ^60^ ^]^ This intermediate played a crucial role in the photocatalytic reduction of CO₂ to CO, as it originated from the activation and reaction of CO₂ adsorbed on the catalyst surface (Figure S34, Supporting Information). Notably, these COOH^*^ peaks were weak in the 2ML CdSe system.

These data suggested that the formation of the COOH^*^ intermediate in the V_Se_‐2ML CdSe sample was responsible for the enhanced CO production during the photocatalytic CO₂ reduction process. Although CO can also be detected in the CO₂ photoreduction process of the 2ML CdSe system, the corresponding in situ FTIR absorption peaks of the COOH intermediate^*^ are relatively weaker (Figure S34, Supporting Information), indicating a lower efficiency of COOH^*^ formation in the 2ML CdSe system.″

In essence, the most obvious difference in the in situ FTIR spectra for these two samples was the CO^*^ absorption peak at 1954 and 2078 cm^−1^could be detectable once the COOH^*^ available in the V_Se_‐2ML CdSe system, also suggesting that abundant CO^*^ species could be generated on the surface of V_Se_‐2ML CdSe, accounting for the selective generation of CO, which also indicated that CO^*^ was produced on the surface of V_Se_‐2ML CdSe according to the reductive elimination assisted by the proton of COOH^*^ intermediate. Furthermore, there would be stronger interactions between the CO_2_/H_2_O and the surface of V_Se_‐2ML CdSe with defects (Se vacancies) and easier electron transfer from V_Se_‐2ML CdSe to CO_2_/H_2_O, which was illustrated already by the DFT calculation (Figure 1; Figure S3, Supporting Information). Therefore, the possible CO_2_ reduction pathway on the V_Se_‐2ML CdSe could be speculated as follows:

(1)

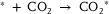

(2) $${{\mathrm{CO}}_{2}}^{*}\;+e-+H+\to \;{\mathrm{COOH}}^{*}$$

(3) $${\mathrm{COOH}}^{*}+e-+H+\to \;{\mathrm{CO}}^{*}\;+\;H_{2}O\;$$

(4)

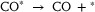

where the "^*^" was denoted adsorption active sites of V_Se_‐2ML CdSe. In other words, the possible pathway of CO_2_ reduction on the V_Se_‐2ML CdSe was that: First of all, CO_2_ was adsorbed on the surface of V_Se_‐2ML CdSe, while H_2_O was decomposed into hydroxy and hydrogen ions. Next, CO_2_^*^ interacted with proton and electron to produce COOH^*^. Then, the COOH^*^ intermediate was reductive elimination, which was assisted by a proton to produce CO^*^. At last, CO^*^ was desorbed to produce CO.

## 7 Conclusion

In conclusion, we have developed a direct and effective surface treatment strategy to introduce defects into NPLs, thereby enhancing solar energy utilization and charge separation efficiency, resulting in highly efficient CO_2_‐to‐CO photoreduction in CdSe NPLs. We discovered that by leveraging the oxidative properties and Lewis acidity of NOBF_4_, defects (Se vacancies) can be controllably introduced on the surface of CdSe NPLs without compromising their structure or morphology. Our findings demonstrate that these defects can act as electron traps, thereby enhancing the photocatalyst's light response by improving the efficiency of photogenerated carrier separation and charge transport. The CdSe NPLs with defects showed enhanced photoreduction CO_2_ performance, reaching as high as 4 folds. The defective CdSe NPLs exhibit superior CO_2_ photo‐oxidation performance, with a CO production rate reaching 2557.5 µmol g^−^¹ h^−^¹ and maintaining stability over 5 h of continuous reaction without significant degradation. The defect introduction strategy proposed in this work provides a novel approach for designing advanced defect‐structured 2D semiconductor photocatalysts with high CO_2_ photoreduction efficiency.

## Conflict of Interest

The authors declare no conflict of interest.

## Supporting information



Supporting Information

## Data Availability

The data that support the findings of this study are available from the corresponding author upon reasonable request.
