# Supplementary material for: Boosted CO2 Photoreduction Performance by CdSe Nanoplatelets via Se Vacancy Engineering
Source: Adv Sci (Weinh). 2025 Feb 7;12(12):2413684. doi: 10.1002/advs.202413684 (PMC11948053; doi:10.1002/advs.202413684)
Supplement: Supplementary file 1 — Supporting Information [file ADVS-12-2413684-s001.pdf]

ADVANCED SCIENCE Open Access

Supporting Information

for *Adv. Sci.*, DOI 10.1002/advs.202413684

Boosted CO_2 Photoreduction Performance by CdSe Nanoplatelets via Se Vacancy Engineering

Huanhuan Luo, Xuanzhao Lu, Yue Cao, Zhaoyuan Lyu, Shichao Ding, Yuehe Lin, Yang Zhou, Wenlei Zhu* and Yuanyuan Wang**

Supporting Information

Boosted CO_2 Photoreduction Performance by CdSe Nanoplatelets via Se Vacancy Engineering

Huanhuan Luo,[1]† *Xuanzhao Lu,* [1]† *Yue Cao,* [1] *Zhaoyuan Lyu,* [3] *Shichao Ding,* [3] *Yuehe Lin* [3] *Yang Zhou,* [2]* *Wenlei Zhu,* [1]* *Yuanyuan Wang,* [1]*

[1]H. Luo, X. Lu, Y. Cao, W. Zhu, Y. Wang

State Key Laboratory of Coordination Chemistry, State Key Laboratory of Pollution Control and Resource Reuse, State Key Laboratory of Analytical Chemistry for Life Science, the Frontiers Science Center for Critical Earth Material Cycling, School of Chemistry and Chemical Engineering, School of the Environment, Nanjing University, Nanjing 210023, China.

[2]Y. Zhou

State Key Laboratory for Organic Electronics & Information Displays, Institute of Advanced Materials, Nanjing University of Posts & Telecommunications, Nanjing 210046, China.

[3]Z. Lyu, S. Ding, Y. Lin

School of Mechanical and Materials Engineering, Washington State University, Pullman, WA, 99164, USA.

*Corresponding authors: Y. Zhou, W. Zhu, Y. Wang; E-mail: iamyangzhou@njupt.edu.cn (Y. Zhou); wenleizhu@nju.edu.cn (W. Zhu); wangyy@nju.edu.cn (Y. Wang).

†The authors contribute equally to this work.

Content

Materials and Methods: 2
Supplementary Figures: 9
Supplementary References: 32

Materials and Methods:

Materials: Cadmium acetate dihydrate ($Cd(OA)_2·2H_2O$), Cadmium oxide (CdO), Cadmium nitrate tetrahydrate ($Cd(NO_3)_2·4H_2O$), 1-Octadecene, Oleic acid (OA), Octanoic acid, propionic acid (PA), Selenium (Se), were obtained from Shanghai Aladdin Biochemical Technology Co., Ltd. Myristic acid (MA), Sodium hydroxide (NaOH), Methanol (MeOH), Ethanol (EtOH), Isopropyl alcohol (IPA), *n*-hexane (Hex), toluene (tol), Acetone, N, N-Dimethylformamide (DMF) were purchased from Sinopharm Chemical Reagent Co., Ltd, China. Nitrosonium tetrafluoroborate ($NOBF_4$) were purchased from Thermo Fisher scientific, Indium nitrate hydrate ($In(NO_3)_3$) were purchased from Sigma-Aldrich LLC. All chemicals were analytically pure and used without further purification.

Preparation of Cadmium Octanate ($Cd(OOCC_7H_{15})_2$): CdO (15.7 mmol, 2.000 g) was dispersed in octanoic acid (40.0 mmol, 5.768 g), and then the suspension was heated to 210 °C from room temperature under N_2 atmosphere and kept 20 min, while it was turn to transparent solution. It was cooled down and degassed at 100 °C laterly. After degassing, it was purified by washing with acetone more than 3 times and collected the white solid to dry.

Preparation of Cadmium Myristate ($Cd(My)_2$): NaOH (6.0 mmol, 0.240 g) and Myristic acid (6.0 mmol, 1.370 g) was well dispersed in MeOH (240.0 mL) and stirred for 1 h. At the same time, $Cd(NO_3)_2·H_2O$ (2.0 mmol, 0.617 g) was well dispersed in MeOH, and then it was droped into the former solution, the white emulsion would be obtained gradually. It was purified by washing with MeOH more than 5 times after 10 h and collected the white solid to dry.

Preparation of TOP-Se-1M: Se shots (1.0 mmol, 78.96 mg) was dispersed in TOP (1.0 mL) and heated until the solution turned to transparent.

Peparation of 2ML CdSe nanoplatelets (NPLs): 2ML CdSe NPL was synthesized according to previous literature without further modifications.[1] In a three-neck round-bottom flask, $Cd(OOCC_7H_{15})_2$ (0.5 mmol, 199.21 mg) and ODE (12.0 mL) were degassed for 1 h at 100 °C. The temperature was set to 160 °C, and PA (100.0 μL), TOP-Se-1M (100.0 μL) and ODE (1.0 mL) were swiftly injected into the flask when it heated to 150 °C. The reaction was kept for 20

min at 160 °C, then OA (2.0 mL) was added. The suspension was cooled to 80 °C to purification. Washing procedure: Hex (20.0 mL) and IPA: EtOH=1: 5 (v:v, 15.0 mL) were added to the suspension. The suspension was centrifuged for 10 min at 4300 rpm. The NPLs precipitated under these conditions, while the byproducts (dots, clusters) remained in the liquid phase. The supernatant was discarded, and the solid was redispersed in *n*-hexane and centrifuged at 4200 rpm to remove the unreacted carboxylates. The solid was discarded, and the supernatant, containing 2ML CdSe NPLs, was collected and stored as *n*-hexane dispersion.

Preparation of 4ML CdSe NPLs: 4ML CdSe NPL was synthesized according to previous literature without further modifications,[2] in a three-neck flask, $Cd(My)_2$ (0.3 mmol, 170.0 mg), Se (0.15 mmol, 12.0 mg), and ODE (15.0 mL) were degassed for 1 h at room temperature. Then, the flask was heated rapidly to 240 °C under nitrogen atmosphere. $Cd(OAc)_2 \cdot 2H_2O$ (0.17 mmol, 46.2 mg) was quickly introduced, when the temperature reached 190 °C. The solution was kept at 240 °C for 1min, and the reaction was then cooled down to stop. OA (1.0 mL) and Hex (15.0 mL) were added when the temperature was reduced to 70 °C. The mixture was then centrifuged at 8000 rpm, the supernatant was discarded and the precipitate containing the NPLs was resuspended in *n*-hexane.

Preparation of 5ML CdSe NPLs: 5ML CdSe NPL was synthesized according to previous literature with minor modifications,[2] in a three-neck flask, $Cd(My)_2$ (0.3 mmol, 170.0 mg), and ODE (14.0 mL) were degassed for 30 min at room temperature. Then, the flask was heated rapidly to 240 °C under nitrogen atmosphere. When the temperature reached 190 °C, ultrasonicated Se-ODE solution (0.15 M, 1.0 mL) was quickly introduced, and 20 s later, $Cd(OAc)_2 \cdot 2H_2O$ (0.26 mmol, 69.4 mg) was quickly introduced. The solution was kept at 240 °C for 10 min, and the reaction was then cooled down to stop immediately. OA (1.0 mL) and Hex (15.0 mL) were added when the temperature was reduced to 70 °C. The mixture was then centrifuged at 9200 rpm, the supernatant was discarded and the precipitate containing the NPLs was resuspended in *n*-hexane.

Preparation of defective 2ML CdSe NPLs (V_{Se}-2ML CdSe also noted as 2ML CdSe-$NOBF_4$ in SI): 2ML CdSe NPL dispersed in Hex (0.9 mg mL^{-1}, 1.0 mL) was added to $NOBF_4$ dispersed in DMF (0.2 mg mL^{-1}). The mixture was hand shaked vigorously for 20 times, and then centrifuged at 12000 rpm for 45s -1min. The supernatant was discarded and the precipitate was resuspended in DMF (400.0 μL) for further purifying, tol: Hex=1: 2 (v: v, 1.2 mL) was added to the solution and then centrifuged at 12000 rpm for 2 min, collect precipitate for repeating the purified procedure again. At last, precipitate V_{Se}-2ML CdSe NPLs was resuspended in DMF. Defective 4ML CdSe NPL (V_{Se}-4ML CdSe also noted as 4ML CdSe-$NOBF_4$) and defective5ML CdSe NPL (V_{Se}-5ML CdSe also noted as 5ML CdSe-$NOBF_4$) were obtained in the same procedure.

Preparation of 2ML CdSe-$In(NO_3)_3$: 2ML CdSe-$In(NO_3)_3$ was obtained in the similar way of 2ML CdSe-$NOBF_4$. 2ML CdSe NPL dispersed in Hex (0.9 mg mL^{-1}, 1.0 mL) was added to $In(NO_3)_3$ dispersed in DMF (1.1 mg mL^{-1}, 1.0 mL)). The mixture was hand shaked vigorously for 20 times, and then centrifuged at 12000 rpm for 45s -1min. The supernatant was discarded and the precipitate was resuspended in DMF (400.0 μL) for further purifying, tol: Hex=1: 2 (v: v, 1.2 mL) was added to the solution and then centrifuged at 12000 rpm for 2 min, collect precipitate for repeating the purified procedure again. At last, precipitate 2ML CdSe-$In(NO_3)_3$ was resuspended in DMF. 5ML CdSe-$In(NO_3)_3$ were obtained in the same procedure.

Preparation of 2ML CdSe-plasma:[3, 4] 2ML CdSe (0.9 mg mL^{-1}, 1.431 mL) was drop cast onto quartz (2 cm x 2 cm) substrate, and then it was heated at 80 °C until the weight unchanged. The quartz substrate with 2ML CdSe was exposure to [Air] plasma (80 Pa, 100 W, 10 min) and then the sample 2ML CdSe-palsma was obtained.

Characterization: The phase compositions of as-prepared samples were characterized by powder XRD (D8 Advance, Bruker, Germany) with Ni-filtered Cu-Kα (λ = 1.540598 Å), while the XRD data was collected in a 2θ range from 20° to 80°. Transmission electron microscope (TEM) (JEM-2800, JEOL, Japan) were employed for the determination of morphological structure of as-prepared samples. The chemical compositions of as-prepared samples were

characterized by XPS electron spectrometer (PHI5000 VersaProbe, ULVAC-PHI, Japan) using 150 W Al Kα X-ray sources. The laser confocal Raman spectrometer was used to collect the Raman scattering information of the sample (Renishaw InviA-Reflex), which can further confirm the chemical composition of as-prepared samples. The elements stoichiometric ratio as-prepared samples were characterized by ICP-OES (ICP-AESPQ9000). The EPR signals of samples were acquired through a Bruker EMX plus-6/1 variable-temperature X-band apparatus. The UV-Vis absorption and UV–Vis diffused reflectance spectra (DRS) of the samples were collected by a UV–Vis–near–IR spectrometer (Carry 5000, Agilent), in which Poly(tetrafluoroethylene) (PTFE) was reference in DRS test. HORIBA FL-3 3D fluorescence spectrometer (HORIBA FL-3) was used for the collection of PL emission spectra and PL lifetime decay curves of the samples under 374 nm excitation wavelength. The photocurrent measurements of the samples were carried out on a CHI 660B (Chenhua, China) electrochemical workstation at room temperature using 0.1 M Na_2SO_4 as electrolyte. The Electrochemical impedance spectroscopy (EIS) of samples were acquired on a CHI 660E (Chenhua, China) electrochemical workstation at room temperature using 0.1 M $Fe(CN)_6K_3$/ $Fe(CN)_6K_4$ as electrolyte. The IR spectrum of nanoclusters was obtained by Thermo Fisher Nicolet 6700. The components of organic matter and inorganic substances in the samples were analyzed by TGA 4000 (PerkinElmer). N_2 adsorption/desorption isotherms were measured using a Micrometritics ASAP 2020 system, PhysiChem iPore 620 Automated Surface Area and Micropore Analyzer, and a BSD-PS4 gas adsorption analyzer.

Transient absorption (TA) measurements: Femtosecond (fs) transient absorption spectroscopy measurements were investigated by a Pharos ultrafast Yb:KGW laser (Light Conversion) with a regenerative amplifier, which produces IR pulses (10 W) centered at 1030 nm wavelength (200 kHz, pulse width < 290 fs). A portion of this beam (80%) was sent through an optical parametric amplifier (OPA, ORPHEUS, Light Conversion) to generate the pump beam of 340 nm for the excitation (pulse duration ~70 fs), and the residual was used as the probe beam. The resulting pump and probe pulses were directed into a TA spectrometer (Harpia, Light Conversion). Both the pump and probe pulses were sent into an optical bench. The probe pulse was focused into a sapphire crystal, generating continuum white light as the probe light.

The frequency of the pump pulse was reduced to 95 Hz, using a chopper, and the absorption with and without pumping light was measured and compared. For TA measurements in the time domain of 4 ns, a time delay of the probe pulse with respect to the pump pulse was regulated by a computer controlled optical delay stage. Pump and probe pulses were focused and overlapped on the sample plane, which was housed in a 1 mm cuvette. The transmitting white-light probe pulses were directed into a photodiode array detector (Kymera, Andor). The resulting absorption differences were calibrated and analyzed using software (Harpia service app, Light Conversion).

Photocatalytic CO_2 Reduction Test:[5] Photocatalytic CO_2 reduction performance of as-prepared samples was tested on an online photocatalytic test system (as shown in **Scheme S1 and Figure S1**) while a 300 W Xenon lamp (full spectrum) was applied as light source. Typically, 1.0 mg of photocatalyst was coated on a sample stage (2 cm x 2 cm) drying overnight and then placed in a sealed photoreactor (30.0 mL in volume) with a quartz light window. The photoreactor was placed in a dark box to eliminate the influence of ambient light. During the dark reaction process, the CO_2 flow (50.0 Sccm, controlled by a flow meter) was first passed through a bubbler containing 30.0 mL of DI water to carry H_2O into the photoreactor for 5 min while removing air at the same time. Besides, the CO_2 flow was set as the standard rate (8 Sccm) for further gas exchange (note: the CO_2 flow rate was changed in the exploring the relationship of rate and CO_2 producing rate). The gas exchange process lasted for more than 30 min to create an anaerobic environment in the photoreactor and to ensure the adsorption of CO_2 and H_2O molecules on the surface of the photocatalyst. Thereafter, the Xenon lamp was turned on to start the photocatalytic CO_2 reduction reactions, while the photoreduced products, together with unreacted CO_2 were brought into a gas chromatography (GC, 8890B, Agilent Technologies, America) at fixed time intervals (15 min) for analysis.

Formula for calculating CO evolution rate: As shown in **Figure S1,** the experimental setup consisted of four main components: a flow meter, a bubbler (containing water), a photoreactor, and a gas chromatograph (GC), all connected by tubing with an inner diameter of approximately 1.6 mm. These components formed a continuous-flow reaction system. The CO_2 gas was first

introduced into the flow meter, and after setting the desired flow rate, it passed through the water-containing bubbler to carry water vapor into the photoreactor, where the catalytic reaction occurred. The gas-phase products generated from the reaction then flow along with the CO_2 stream into the GC, equipped with two sample loops with total volumes of 3 mL. The gas flow direction was indicated by green arrows in **Figure S1**.

The two sample loops in the GC were crucial for data analysis. The continuous CO_2 flow transported the reduction products into the sample loops. Before injection, valve 1 (V1) remained closed, and the gas flowed through the sample loops and exited via the "sample out" port of V1. During this process, the gas flow path was as indicated by the red arrows in **Figure S2**. Specifically, the gas entered the GC through the sample inlet and followed this route (**Figure S2a**): sample in → 1 → 6 → sample loop 1 (1 mL) → 3 → 2 → V1. Within V1, the gas flow path was: 10 → 1 → sample loop 2 (2 mL) → 8 → 9 → sample out. Thus, the sample loops remained filled with gas at all times. When an injection command was triggered, V1 and V3 rapidly switched, allowing the sample from the loops to be injected into the GC for analysis. As an example of sample flow through the V1 , the flow path was: sample from the loop 2 → 1 → 2 → column 1 (Col #1) → 6 → 5 → column 2 (Col #2) → other columns → detectors (FID and TCD) (**Figure S2b**). The gas components were then analyzed by the detectors.

Based on the description above, the GC sample loops functioned as gas reservoirs during the reaction process, providing sufficient sample volume for CO content analysis. As a result, the experiment can be sampled and analyzed at specific intervals (e.g., every 15 or 30 minutes). Since CO_2 entered the GC at a constant flow rate, the composition of the sample in the loops changed continuously. Therefore, the CO reduction products detected by the GC represented the instantaneous CO evolution rate from the CO_2 reduction reaction. Our flow reaction system was similar to those reported in the literature,[6] and we used the reported CO evolution rate calculation formula (eq (1)). Since the CO evolution rate in our system was correlated with the CO_2 feed flow rate, we mathematically derived a modified version of eq (1), which included the CO_2 flow rate term (v, in mL min^{-1}), resulting in eq (2). In our actual experiments, the CO evolution rate was calculated using eq (2).

We recognize that the applicability of eq (2) was based on the assumption that CO_2 enters the photoreactor at a fixed flow rate and that the catalytic reaction rate remained constant. This assumption can be validated through GC data analysis. Under this assumption, the

instantaneous CO evolution rate was close to the average evolution rate over time, indicating a stable reaction output rate. Therefore, using eq (2) to calculate the CO evolution rate was both reasonable and reliable.

$$\text{Total CO yield } (\mu\text{mol g}^{-1}) = \frac{(C_{final}\text{-}C_{initial}) \text{ x volumetric flow of product gas}}{\text{Amount of photocatalyst used (g)}} \quad (1)$$

$$\text{Total CO yield } (\mu\text{mol g}^{-1}\text{ h}^{-1}) = \frac{(C_{final}\text{-}C_{initial}) \times \text{volumetric flow of product gas} \times \text{v}}{\text{Amount of photocatalyst used (g)}} \quad (2)$$

In situ Fourier transform infrared measurements: The surface interaction between 2ML CdSe-$NOBF_4$ and CO_2/H_2O at molecular level was investigated by in situ infrared spectroscopy. All IR spectra were recorded on a IR measurement on a Bruker Tensor 27 infrared spectrometer (Bruker, Germany) equipped with an horizonal attenuated total reflectance (HATR) cell (Pike, Madison, WI). The spectra were displayed in absorbance units and acquired with a resolution of 4 cm^{-1}, using 64 scans. For measurement, catalyst (V_{Se}-2ML CdSe or 2ML CdSe NPLs) was initially filled into the sample cup (model HVC-DRP-5, Harrick Scientific, Pleasantville, NY) of the DRIFT system containing an external 300 W Xe lamp, in which the dome of the DRIFTS cell has two KBr windows allowing IR transmission and a third (quartz) window allowing transmission of irradiation. Before the adsorption process, high purity Ar gas of 8.0 mL min^{-1} was purged for 30 min to clean the photocatalyst surface, and then the gas mixture of 8.0 mL min^{-1} CO_2 and H_2O steam was introduced into the reaction chamber. In the dark, CO_2 was adsorbed on the photocatalyst surface for 30 min, the background spectrum in the presence of the V_{Se}-2ML CdSe or 2ML CdSe sample was collected, and then the photocatalyst was irradiated using a 300 W Xe lamp for 60 min. The FT-IR spectra were recorded every two minutes under visible light irradiation in the scanning range of 4000-600 cm^{-1}.

Calculation details: DFT calculations are performed by the Vienna Ab initio Simulation Package (VASP)[7] with the projector augmented wave (PAW) method.[8] The exchange-functional is treated using the generalized gradient approximation (GGA) with Perdew-Burke-Ernzerhof (PBE)[9] functional. The energy cutoff for the plane wave basis expansion was set to 500 eV. Partial occupancies of the Kohn−Sham orbitals were allowed using the Gaussian smearing method and a width of 0.05 eV with spin-orbit coupling. For k-space sampling, $k_1 \times k_2 \times k_3$ Γ-centered Monkhorst-Pack meshes were used, where k_n (n = 1,2,3) was prepared as the

mesh spacing near ($2\pi \times 0.04Å^{-1}$) to each direction. The self-consistent calculations apply a convergence energy threshold of 10-4 eV, and the force convergency was set to 0.05 eV/ Å.

Apparent quantum yield: The apparent quantum yield (AQY) was calculated as the ratio between the number of photogenerated electrons consumption and the number of incident photons, by taking into account the fact that two electrons are required to produce one CO (eq (3)). The wavelength-dependent apparent quantum yield was measured under the same photocatalytic reaction condition, except for the monochromatic light wavelengths (350, 380, 400, 430, 460, 500 nm). All the experiments were repeated at least 2 times in parallel to obtain an average value.

AQY (%) = [2 × number of evolved CO molecules / number of incident photons] × 100% (3)

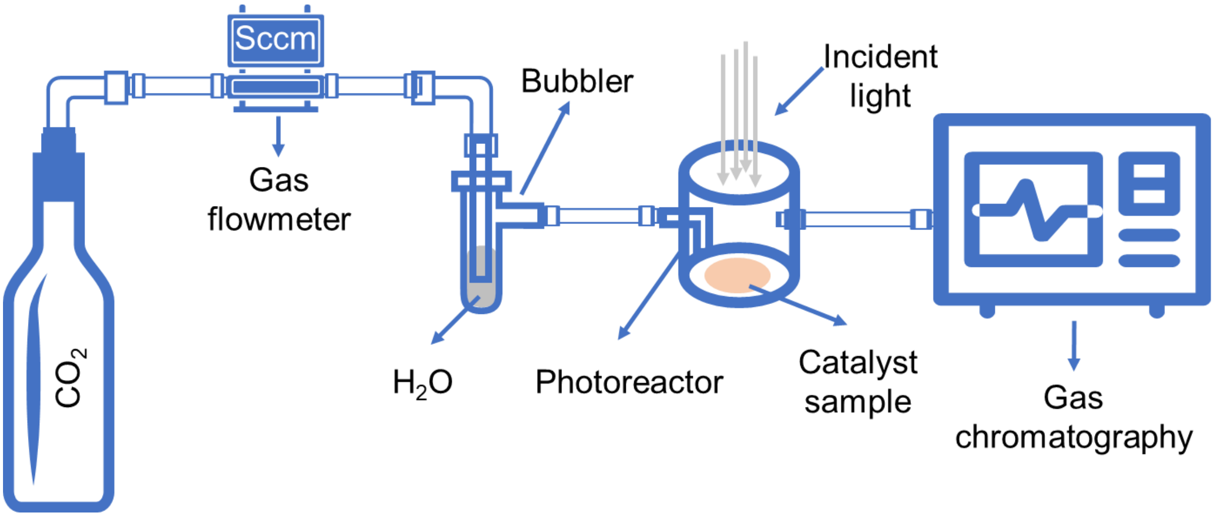

Scheme S1. Illustration of the online photocatalytic test system.

Supplementary Figures:

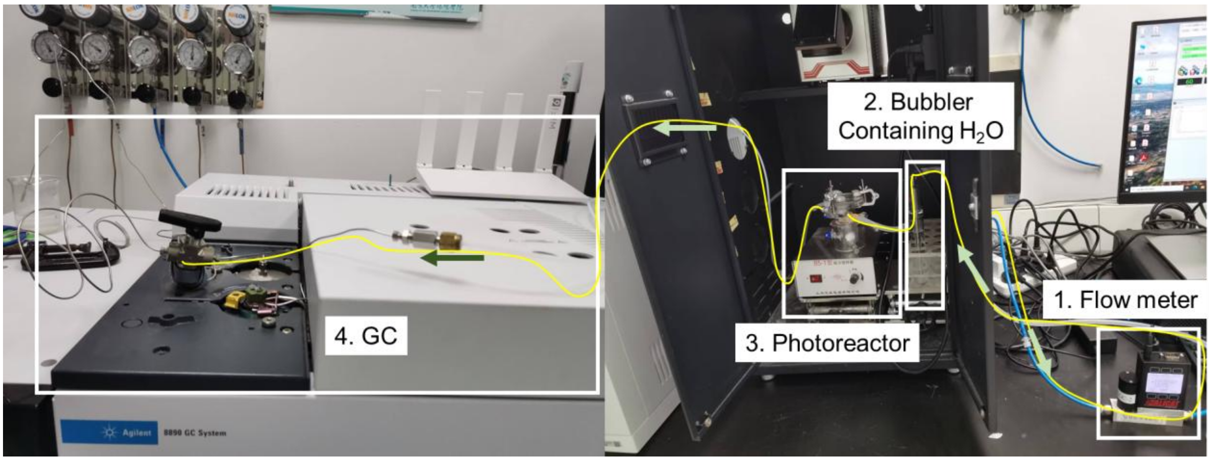

Figure S1. Photograph of CO_2 photoreduction device.

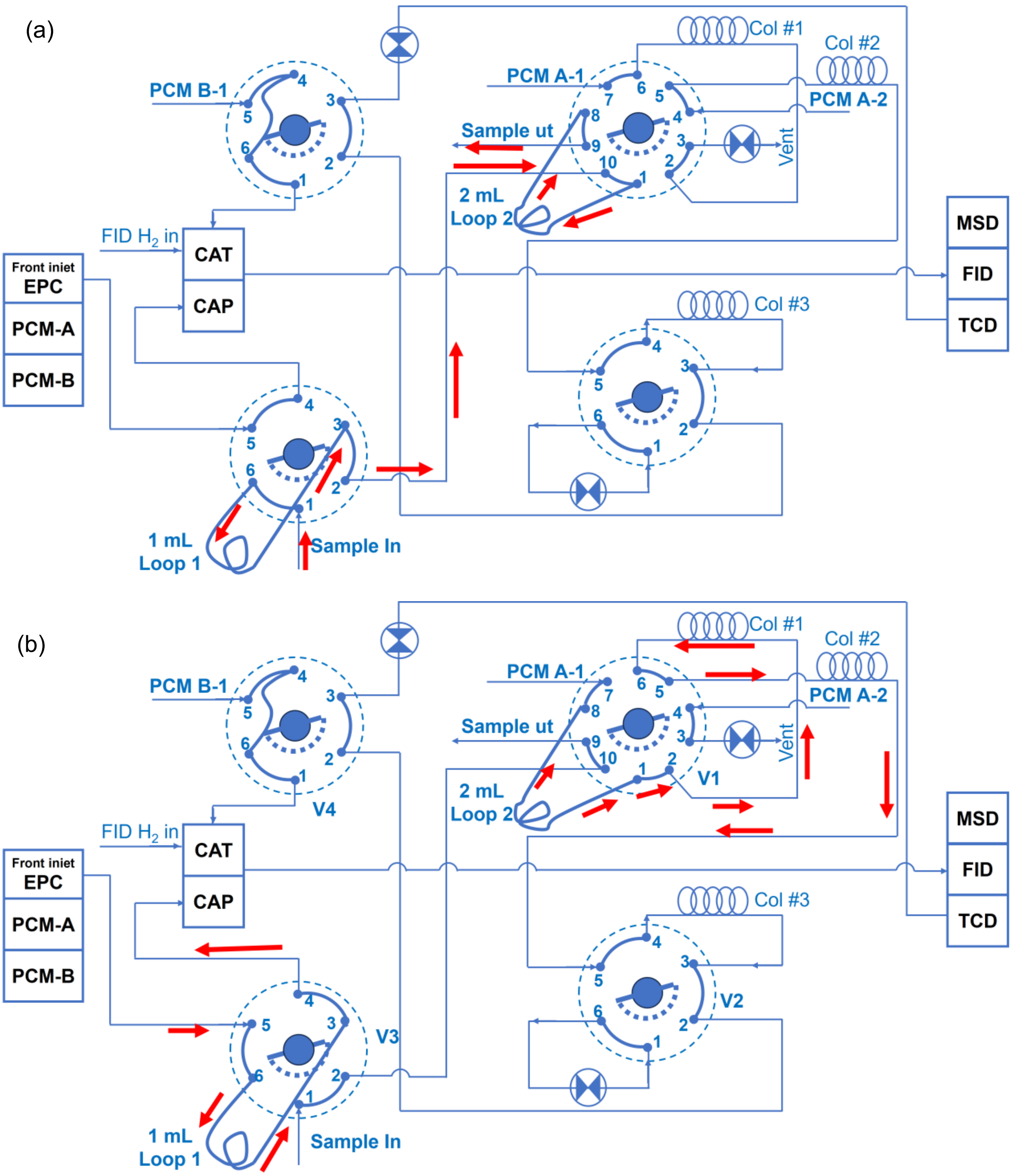

Figure S2. GC valve diagram of gas chromatography. The route of gas flow in GC (a) without injection command, (b) after injection command was triggered. (The blue curve between numbers in the valve section represents the connection. The red arrow represents the direction of gas flow).

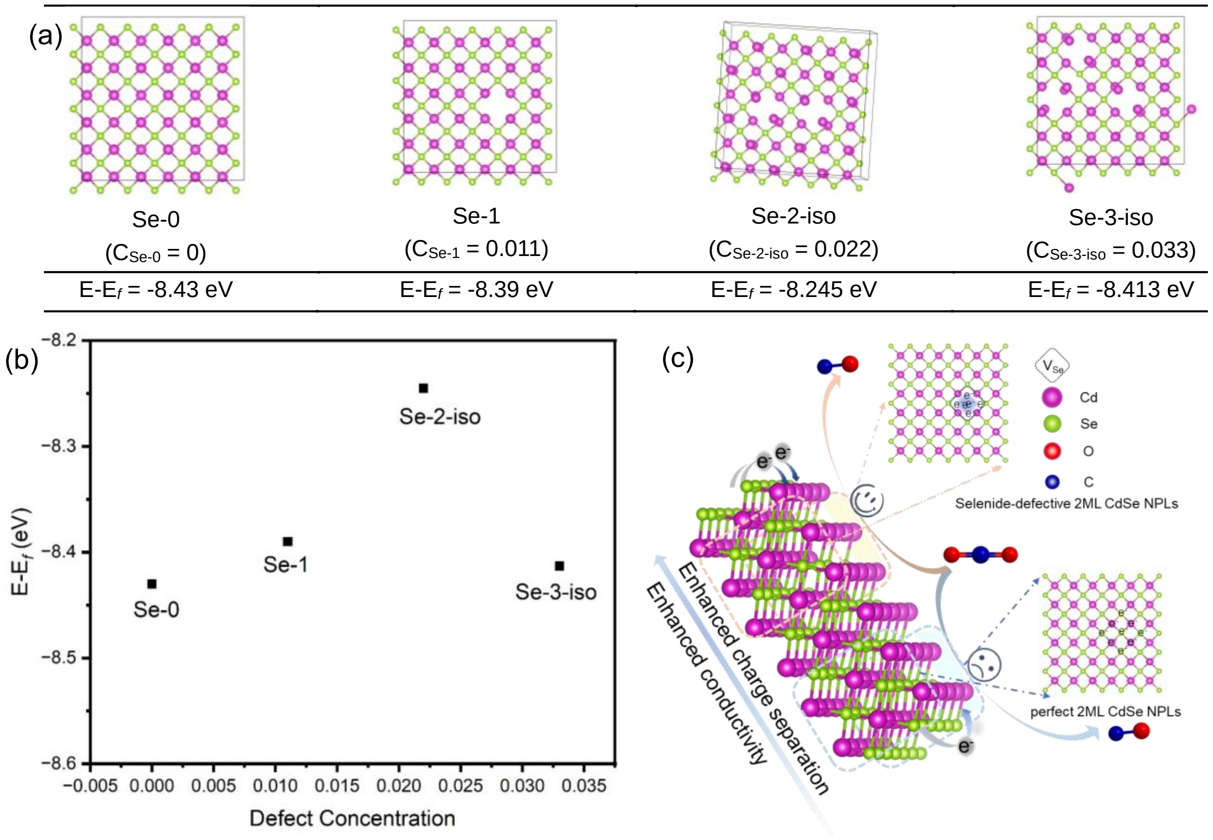

Figure S3. Theoretical study on charge distribution and separation in 2ML CdSe nanoplatelets (NPLs) with selenide vacancies (V_{Se}). (a) The density of states (DOS) for 2ML CdSe NPLs and V_{Se}-2ML CdSe NPLs models in different defect concentration. (b) Trends of d-band center of V_{Se}-2ML CdSe NPLs in different defect (Se vacancies) concentration. More electrons around Fermi Level for V_{Se}-2ML CdSe NPLs than for defectless perfect 2ML CdSe NPLs. (c) Schematic illustration of the possible charge separation and distribution in perfect 2ML CdSe NPLs and selenide-defective V_{Se}-2ML CdSe NPLs models. The electron should be more delocalized between Cd and Se atoms for V_{Se}-2ML CdSe than for 2ML CdSe, along with enhanced charge separation and conductivity due to the Se deficiency, facilitating CO_2 photoconversion to CO. Colour legends: purple, Cd; green, Se; red, O; blue, C.

The detailed modeling process is as follows: We constructed a perfect initial 2ML CdSe unit cell (Se-0) comprising 54 Cd atoms and 36 Se atoms, resembling the structure of a 2ML CdSe nanosheet. Based on this model, we systematically introduced Se vacancies by removing 1 Se atom (Se-1), 2 Se atoms (Se-2-iso), and 3 Se atoms (Se-3-iso), where "iso" represents isolated vacancies, indicating no additional interactions between the vacancies. Following these models, we calculated the d-band center for each sample.

The defect concentration (C_{Se}) was calculated by this equation (4) (eq (4)).

$$C_{Se} = \frac{\text{Number of removing Se atoms}}{\text{Total number of atoms in initial unit cell}} \quad (4)$$

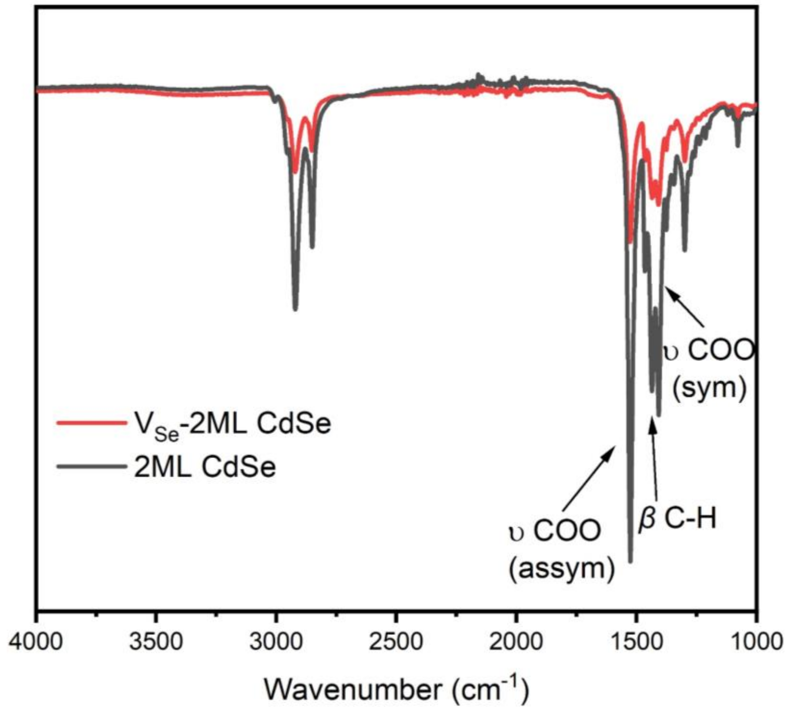

Figure S4. FTIR spectra of 2ML CdSe and V_{Se}-2ML CdSe.

The detailed procedure for FTIR characterization is as follows: We first drop-casted the 2ML CdSe sample onto a KBr pellet and recorded its FTIR spectrum. Immediately after the initial measurement, the sample was immersed in an $NOBF_4$ solution in DMF for approximately 1 minute, followed by rapid drying and subsequent IR measurement. Throughout this process, no additional washing steps were performed on the sample to ensure that sample preparation differences would not contribute to signal weakening in the IR spectra. Each peak in the 1000 cm^{-1} to 4000 cm^{-1} wavenumber range were assigned to specific vibrational modes, indicating the characteristic absorption of cadmium oleate.[10] Considering the ligand-stripping mechanism proposed in our study and supported by the literature,[11] the observed full-band intensity reduction in the FTIR spectra after in-situ $NOBF_4$ treatment aligned with the expected outcome. This decreases in signal across all wavenumbers further confirmed the removal of surface ligands.

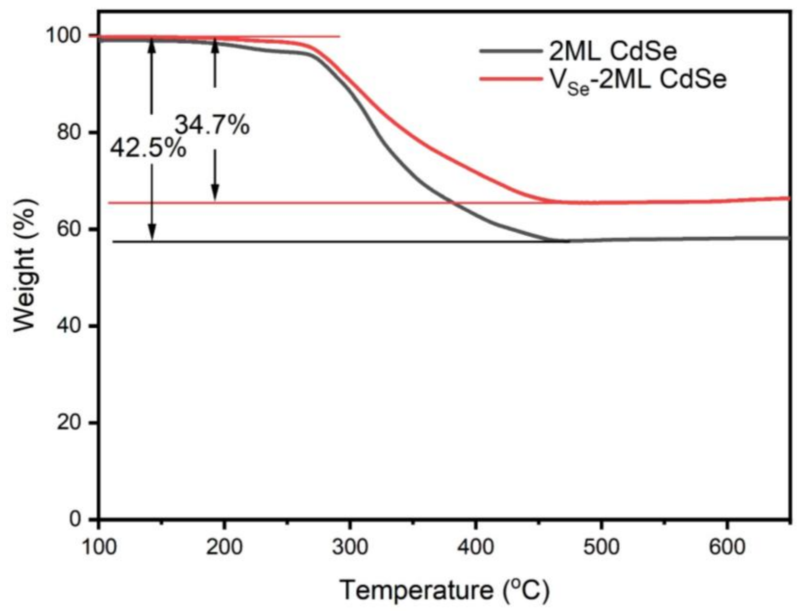

Figure S5. TGA plot of 2ML CdSe and V_{Se}-2ML CdSe.

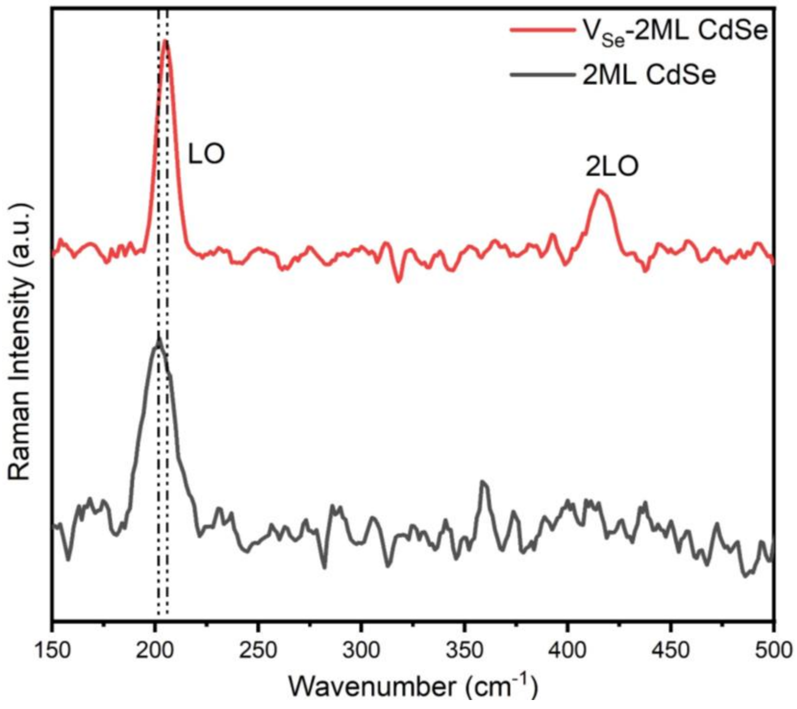

Figure S6. Raman spectra of 2ML CdSe and V_{Se}-2ML CdSe.

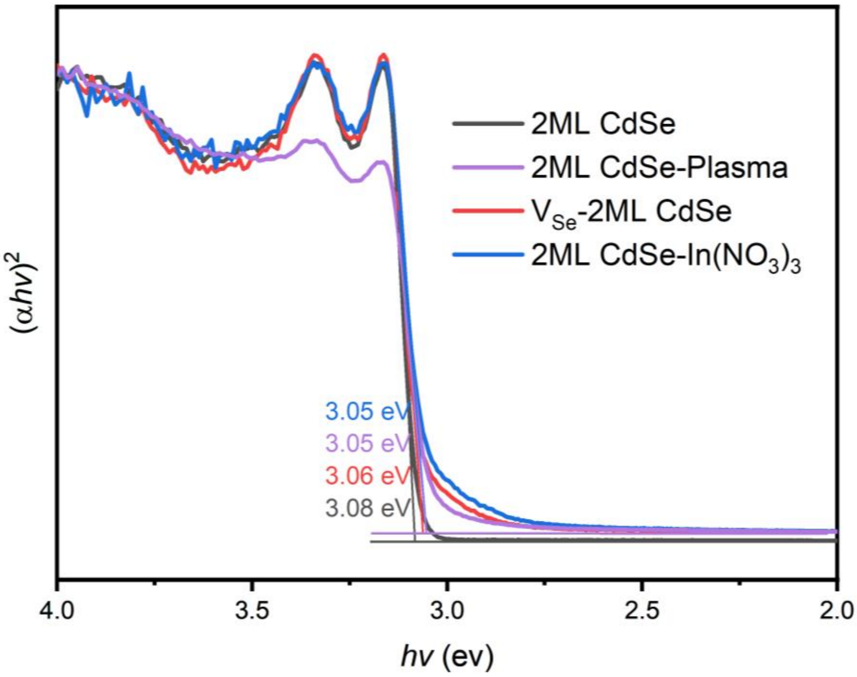

Figure S7. UV-Vis-DRS plots of 2ML CdSe, V_{Se}-2ML CdSe, 2ML CdSe-In$(NO_3)_3$ and 2ML CdSe-Plasma. Note: 2ML CdSe-In$(NO_3)_3$ means introducing Se vacancies to 2ML CdSe by treatment with In$(NO_3)_3$ agent and 2ML CdSe-plasma means introducing Se vacancies to 2ML CdSe by treatment with [air]-plasma.

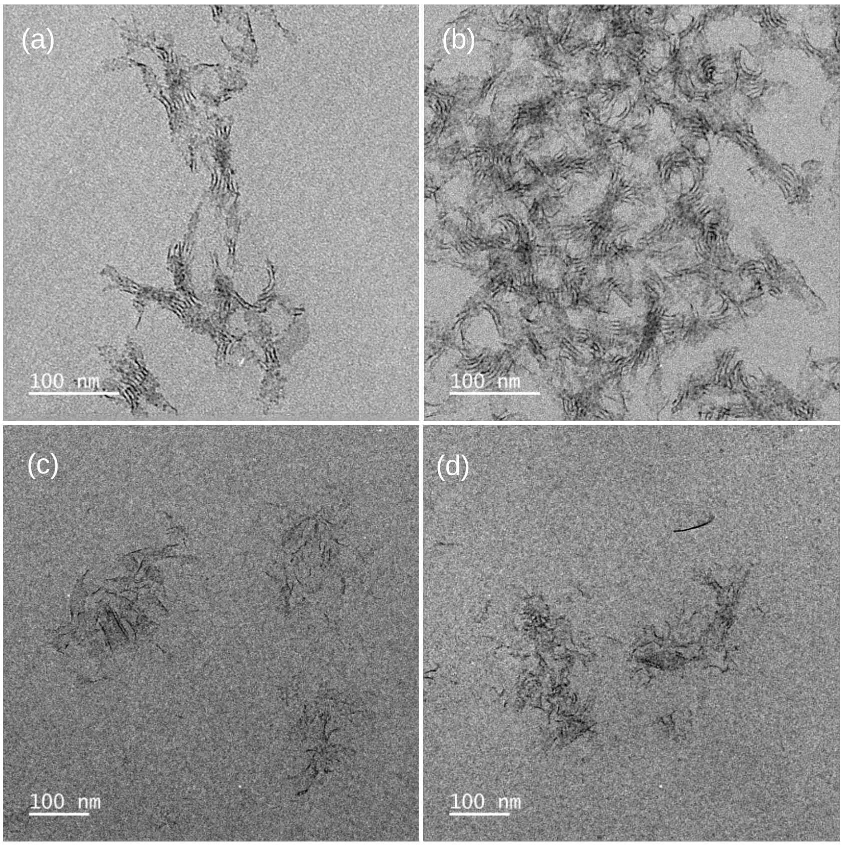

Figure S8.TEM images of (a and b) 2ML CdSe-In$(NO_3)_3$ and (c and d)2ML CdSe-Plasma.

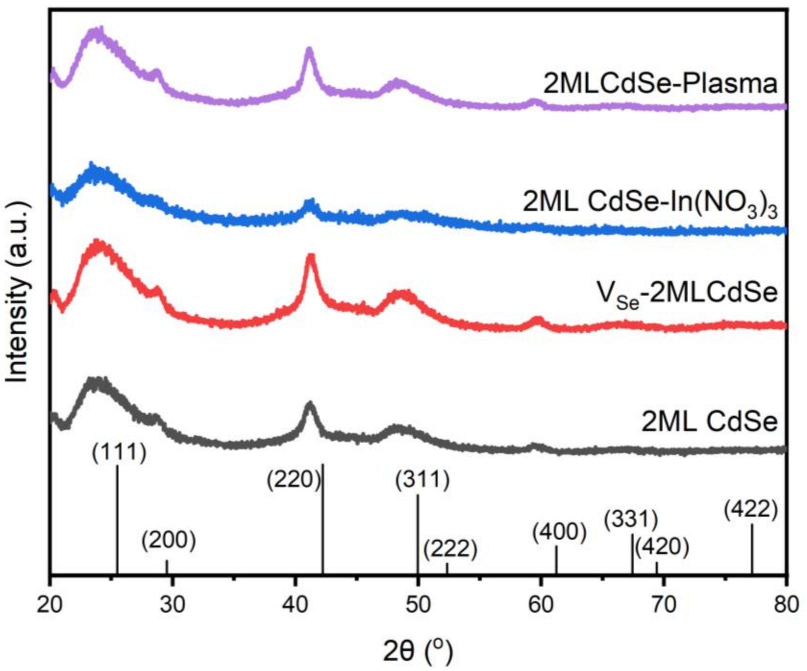

Figure S9. XRD patterns of 2ML CdSe, V_{Se}-2ML CdSe, 2ML CdSe-In$(NO_3)_3$ and 2ML CdSe-Plasma.

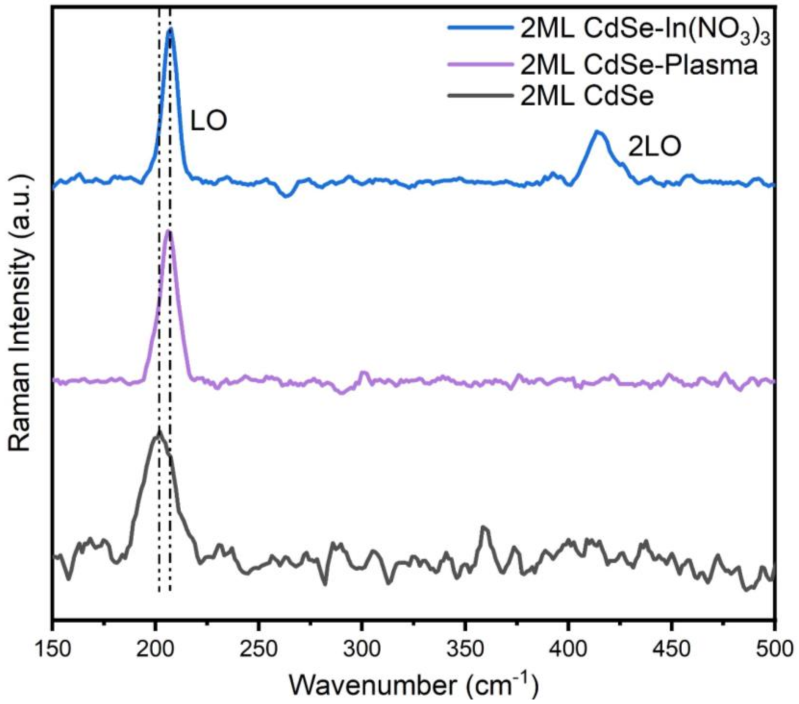

Figure S10. Raman spectrum of 2ML CdSe, 2ML CdSe-In$(NO_3)_3$ and 2ML CdSe-Plasma.

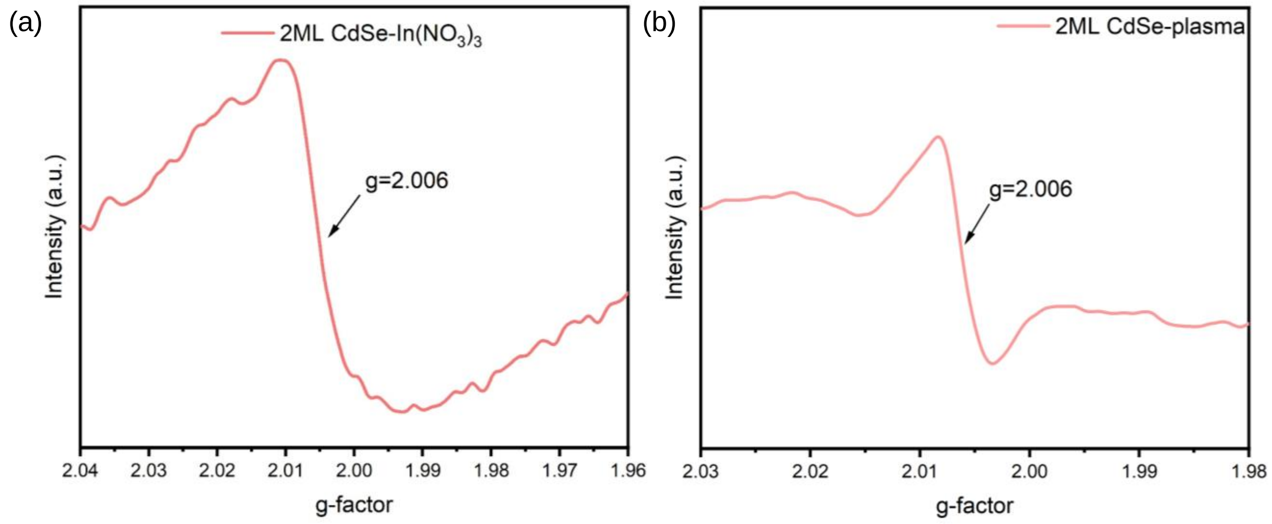

Figure S11. EPR signal of (a) 2ML CdSe-In$(NO_3)_3$, (b) 2ML CdSe-plasma.

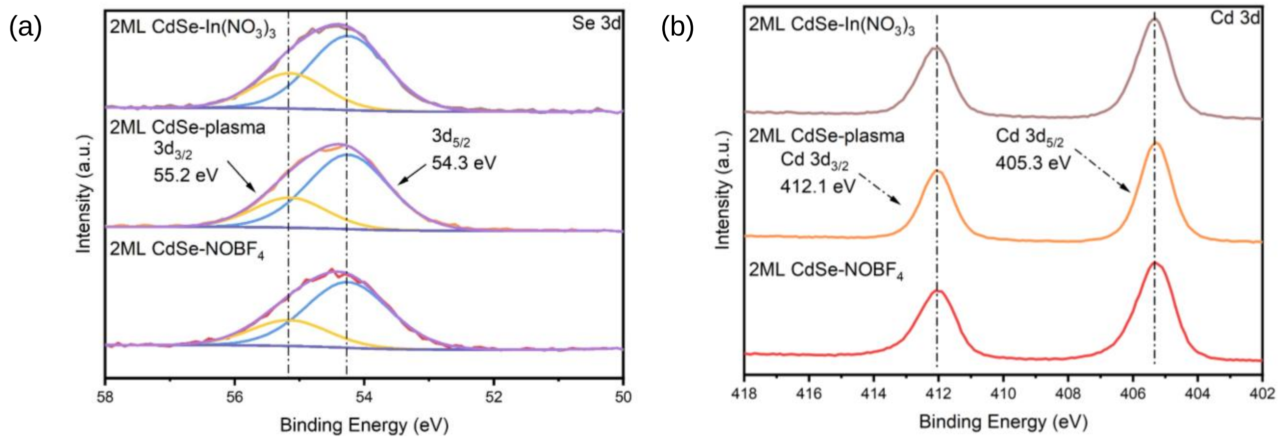

Figure S12. High resolution XPS spectra of (a) Se 3d and (b) Cd 3d of V_{Se}-2ML CdSe (2ML CdSe-$NOBF_4$), 2ML CdSe-plasma, and 2ML CdSe-In$(NO_3)_3$.

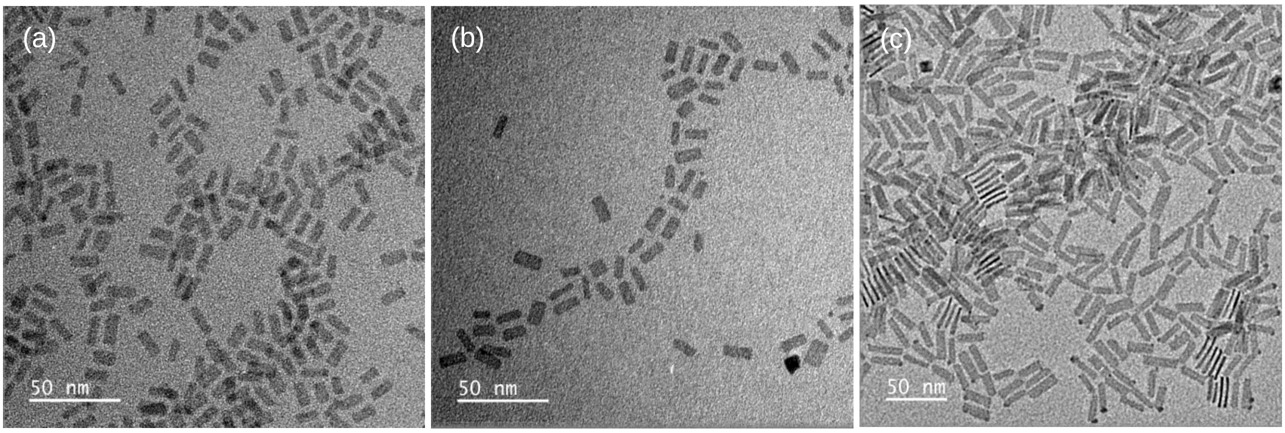

Figure S13. TEM images signal of (a) 5ML CdSe, (b) V_{Se}-5ML CdSe and (c)5ML CdSe-In$(NO_3)_3$.

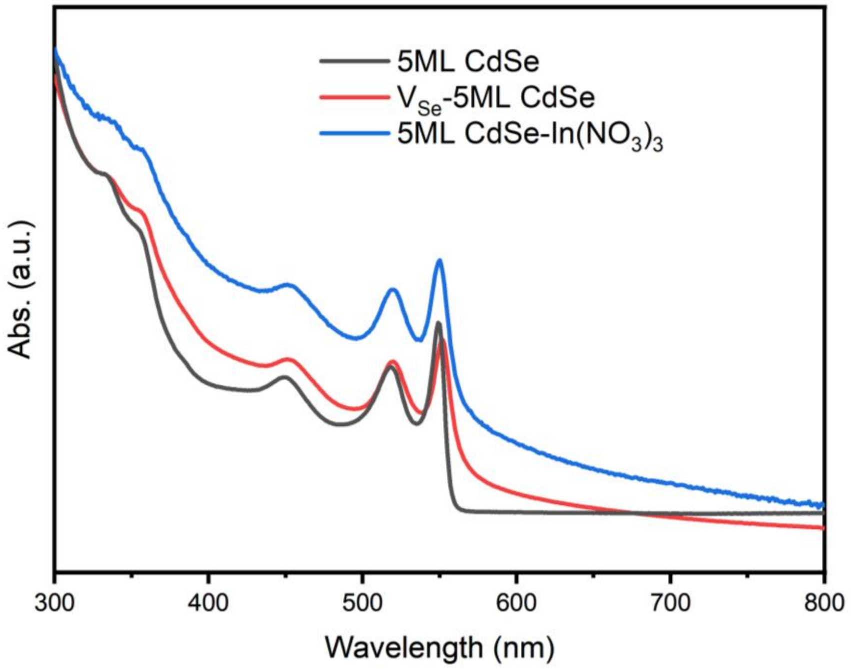

Figure S14. UV-Vis spectra of 5ML CdSe, V_{Se}-5ML CdSe and 5ML CdSe-In$(NO_3)_3$.

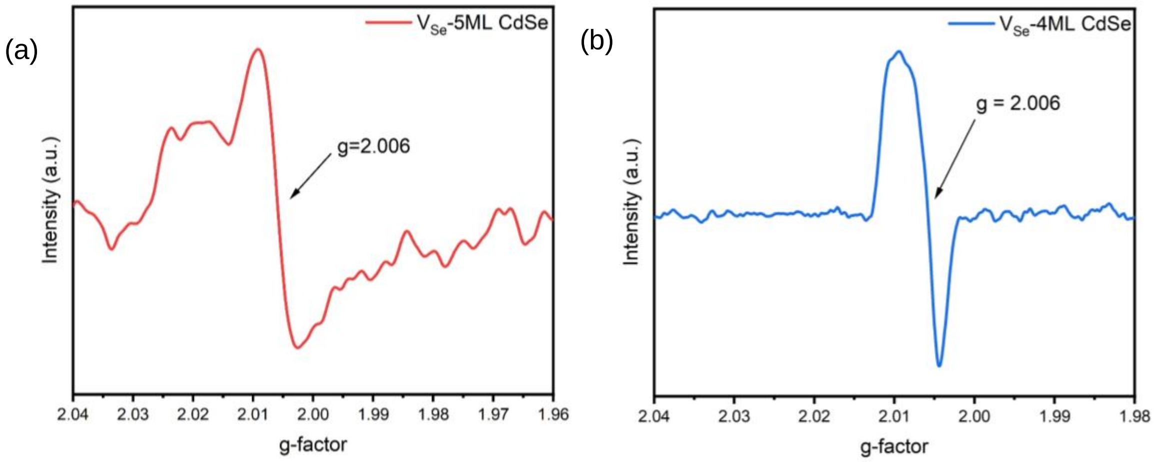

Figure S15. EPR signal of (a) V_{Se}-5ML CdSe and (b) V_{Se}-4ML CdSe.

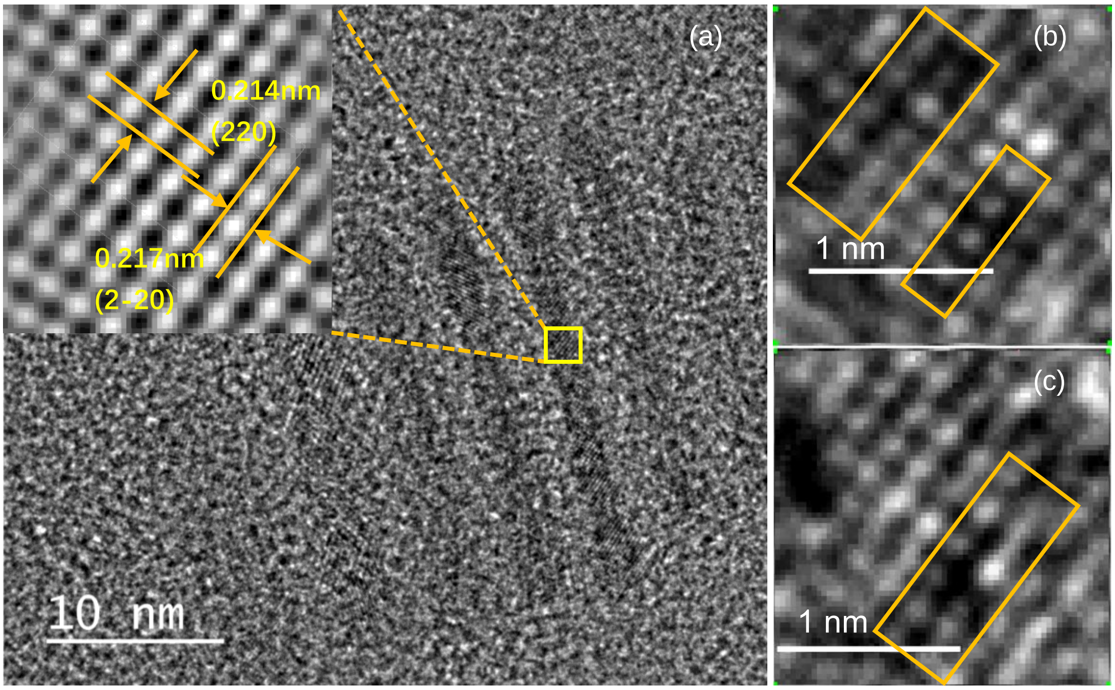

Figure S16. (a) HR-TEM image, inset corresponds FFT pattern and (b and c) the atomic scale of the crystalline phase of 5ML CdSe-$(NO_3)_3$, the parts marked by the orange rectangle are vacancies.

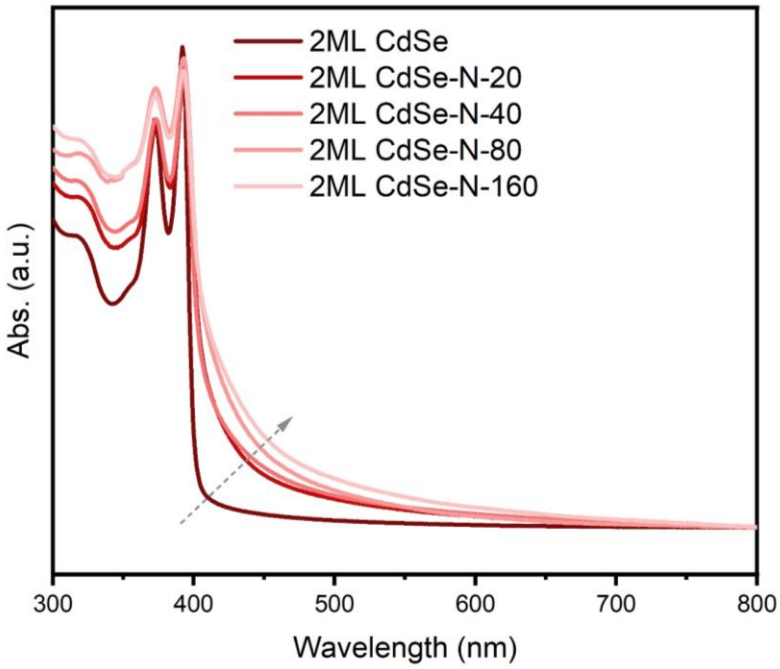

Figure S17. UV-Vis spectra of different V_{Se}-2ML CdSe (2ML CdSe treated with different $NOBF_4$ concentration, the number 20 represent $NOBF_4$ (0.2 mg mL^{-1}) in DMF, 40 represent $NOBF_4$ (0.4 mg mL^{-1}) in DMF, 80 represent $NOBF_4$ (0.8 mg mL^{-1}) in DMF, 160 represent $NOBF_4$ (1.6 mg mL^{-1}) in DMF).

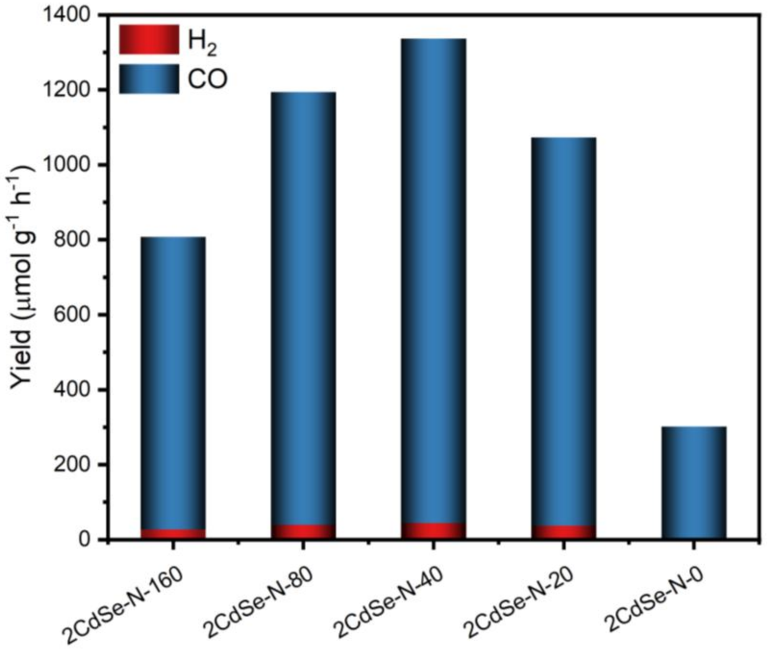

Figure S18. Photoreduction of CO_2 into CO in on-line system over different V_{Se}-2ML CdSe samples (2ML CdSe treated with different $NOBF_4$ concentration).

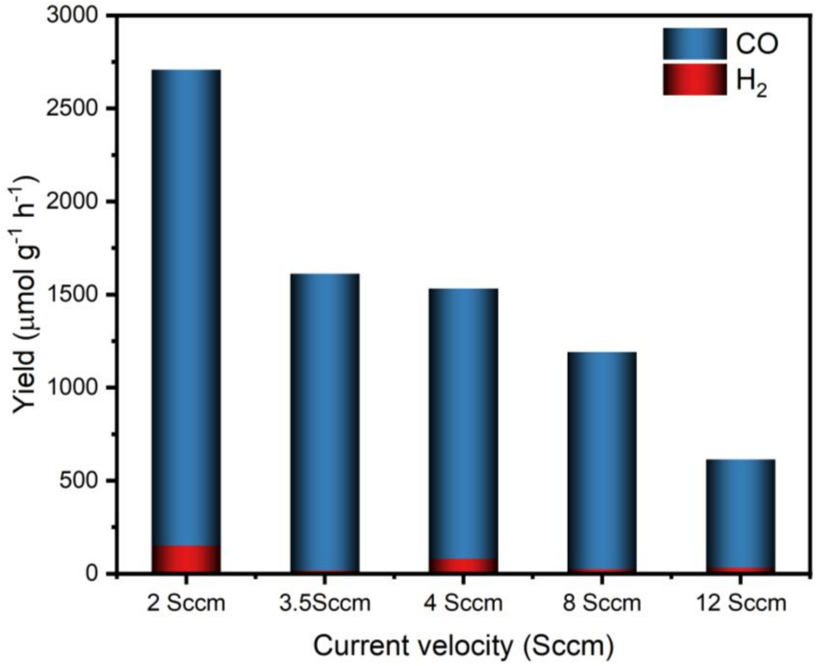

Figure S19. Photoreduction of CO_2 into CO in on-line system under different velocity of CO_2 under the V_{Se}-2MLCdSe samples.

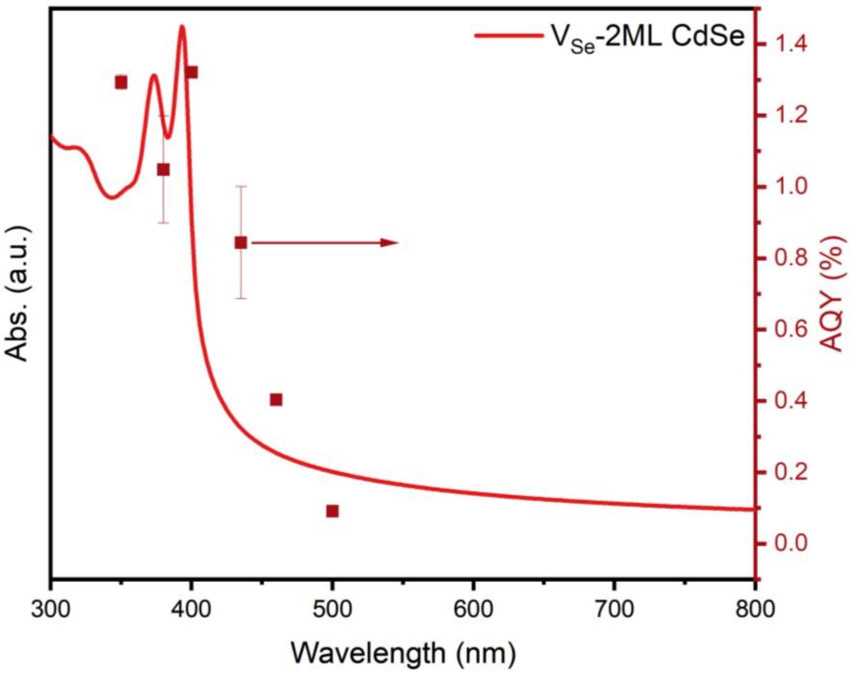

Figure S20. Apparent quantum yields (AQY) together with the UV-Vis spectra of V_{Se}-2ML CdSe.

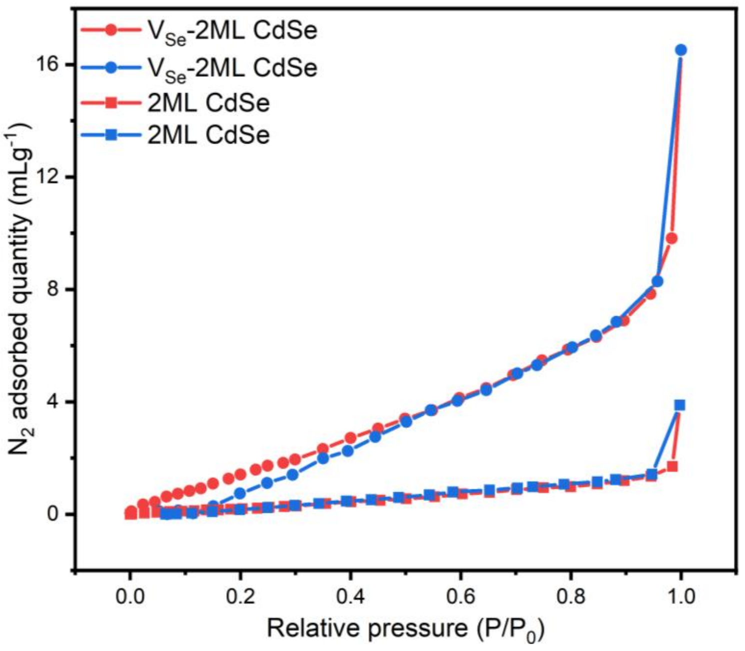

Figure S21. N_2 adsorption-desorption isotherms for V_{Se}-2ML CdSe and 2ML CdSe.

The Brunauer, Emmett and Teller (BET) specific surface area of V_{Se}-2ML CdSe and 2ML CdSe B is 5.9 m^2 g^{-1}, 0.9 m^2 g^{-1}, respectively. Though, V_{Se}-2ML CdSe (5.9 m^2 g^{-1}) processed a slightly larger BET surface area than 2ML CdSe (0.9 m^2 g^{-1}), in some extent, it can be concluded that the contribution of specific surface area to their photocatalytic activity difference can be ignored.

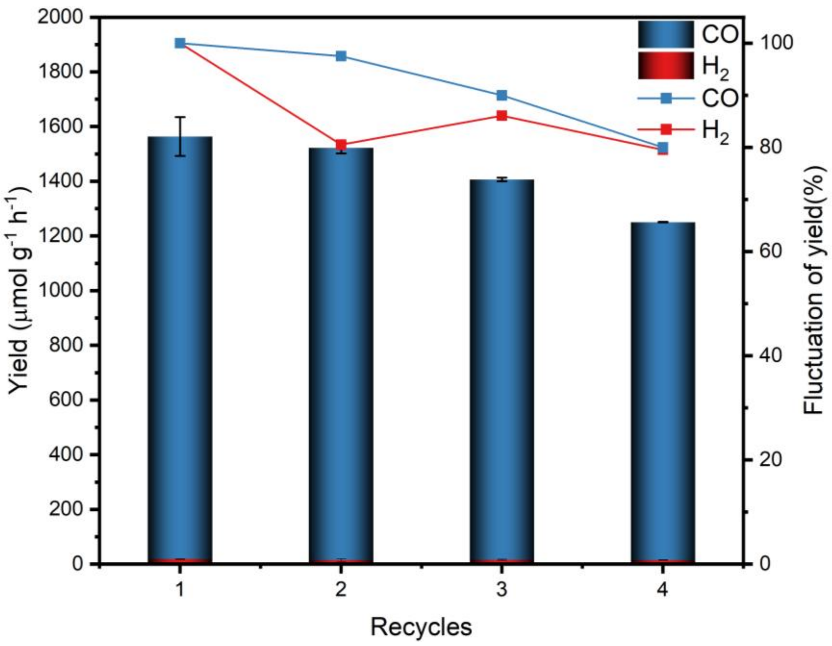

Figure S22. Stability of V_{Se}-2ML CdSe during the photocatalytic CO_2 reduction.

Reaction conditions: Photocatalytic CO_2 reduction performance of V_{Se}-2ML CdSe samples was tested on an online photocatalytic test system (as shown in Scheme S1) while a 300 W Xenon lamp (full spectrum) was applied as light source with CO_2 flow rate of 3.5 Sccm. When the Xenon lamp was turned on to start the photocatalytic CO_2 reduction reactions, while the photoreduced products, together with unreacted CO_2 were brought into a gas chromatography at fixed time intervals (15 min) for analysis. After each cycle, the light irradiation was removed with no catalyst washing, while keeping CO_2 flow.

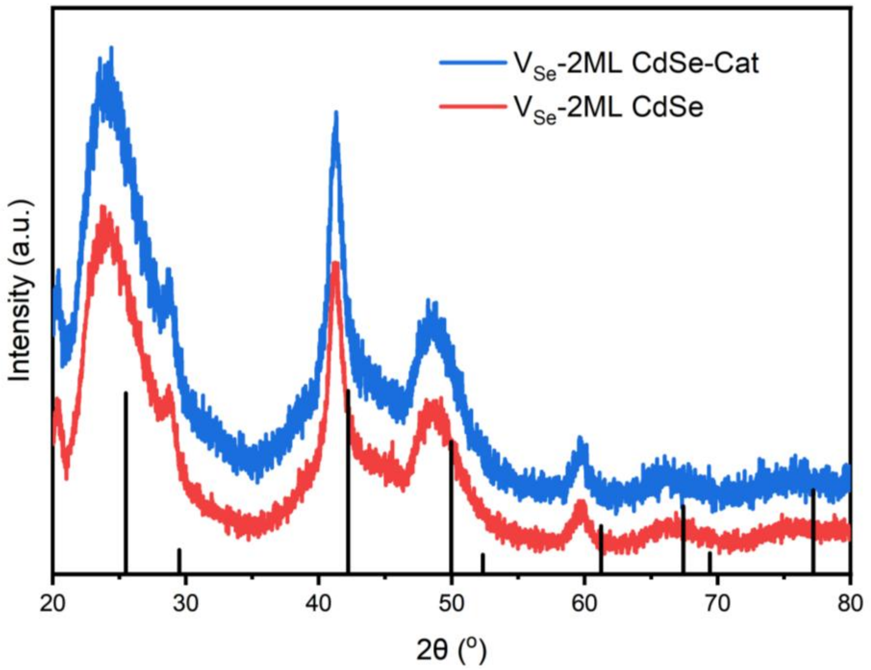

Figure S23. XRD pattern of V_{Se}-2ML CdSe and V_{Se}-2ML CdSe after catalysis (V_{Se}-2ML CdSe-Cat).

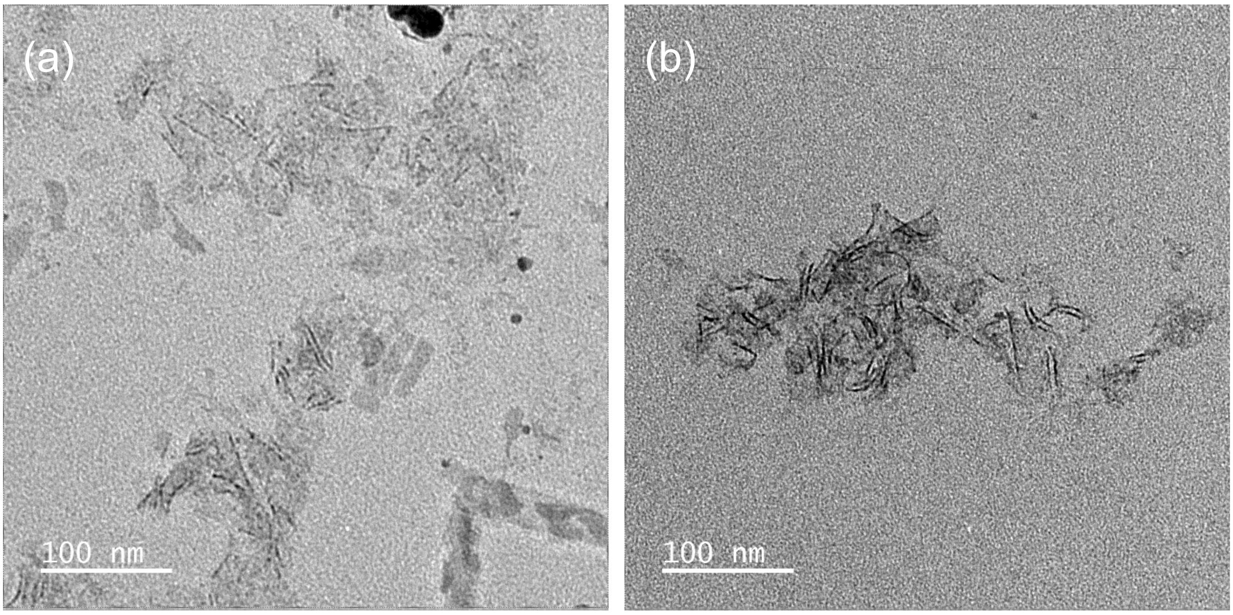

Figure S24. (a and b) TEM images of V_{Se}-2ML CdSe after catalysis (V_{Se}-2ML CdSe-Cat).

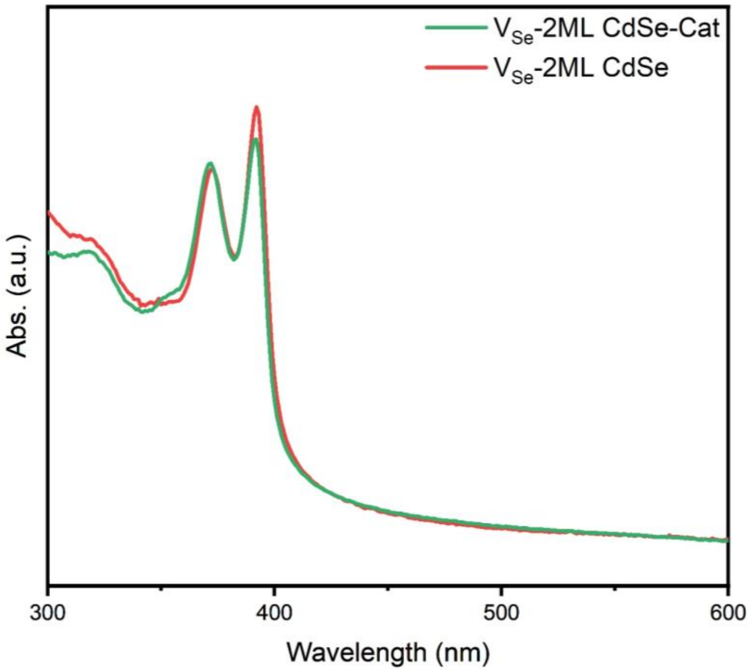

Figure S25. UV-Vis spectra of V_{Se}-2ML CdSe and V_{Se}-2ML CdSe after catalysis (V_{Se}-2ML CdSe-Cat).

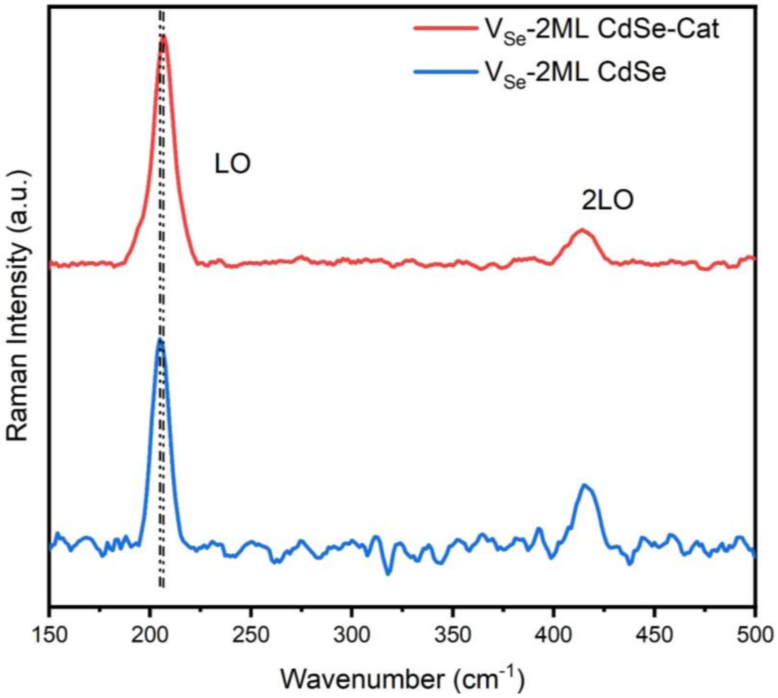

Figure S26. Raman spectra of V_{Se}-2ML CdSe and V_{Se}-2ML CdSe after catalysis (V_{Se}-2ML CdSe-Cat).

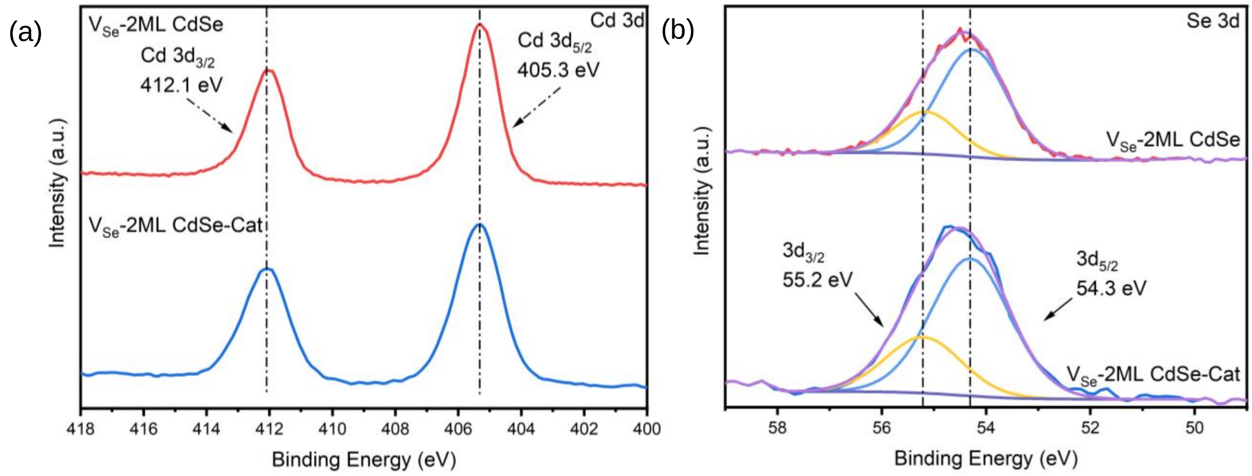

Figure S27. High resolution XPS spectra of (a) Cd 3d and (b) Se 3d in V_{Se}-2ML CdSe, V_{Se}-2ML CdSe-Cat, respectively.

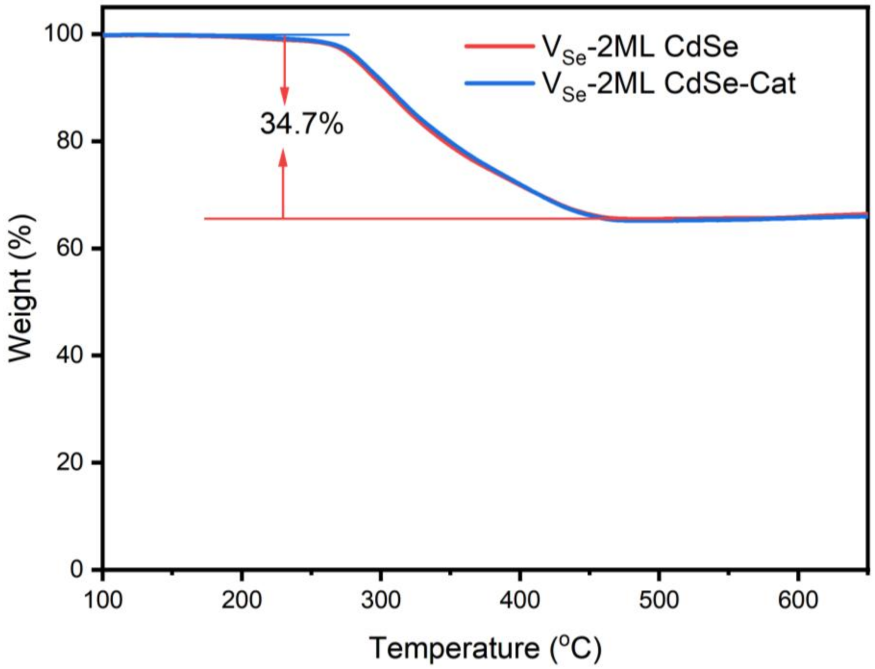

Figure S28. TGA plot of V_{Se}-2ML CdSe and V_{Se}-2ML CdSe after catalysis (V_{Se}-2ML CdSe-Cat).

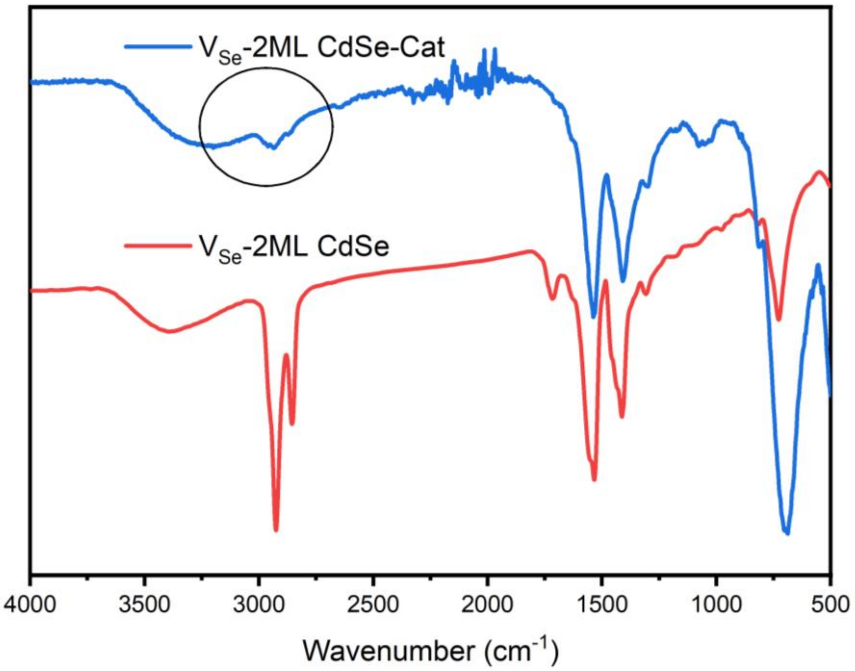

Figure S29. FTIR spectra of V_{Se}-2ML CdSe after catalysis (V_{Se}-2ML CdSe-Cat).

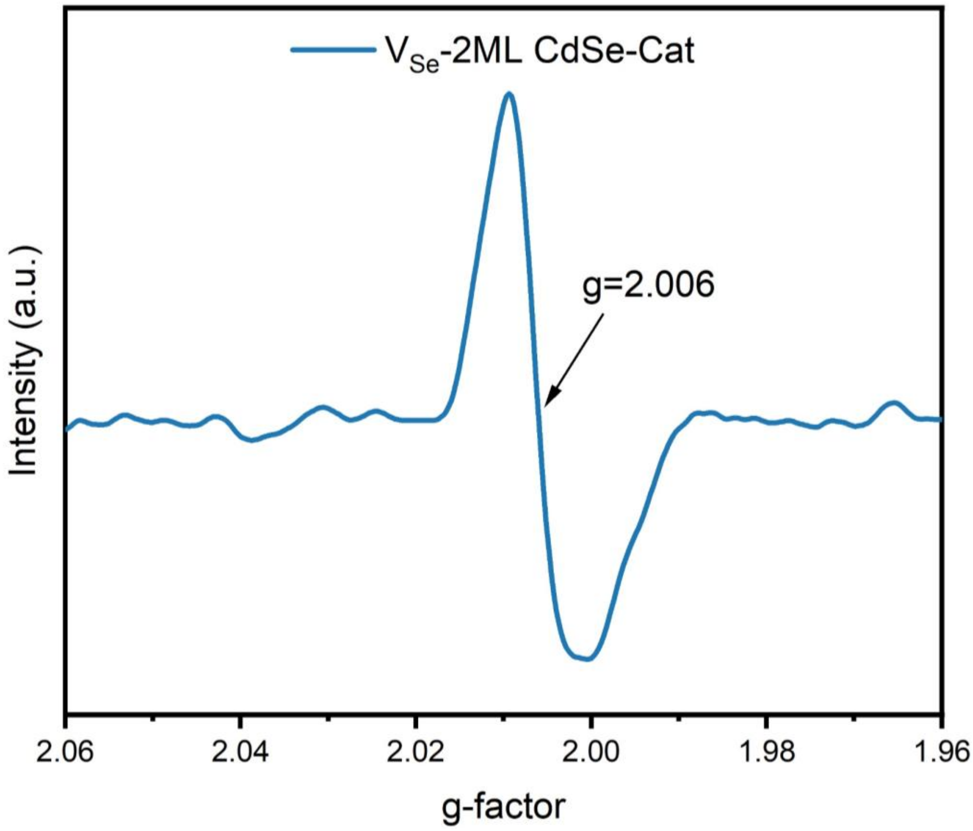

Figure S30. EPR signal of V_{Se}-2ML CdSe after catalysis (V_{Se}-2ML CdSe-Cat).

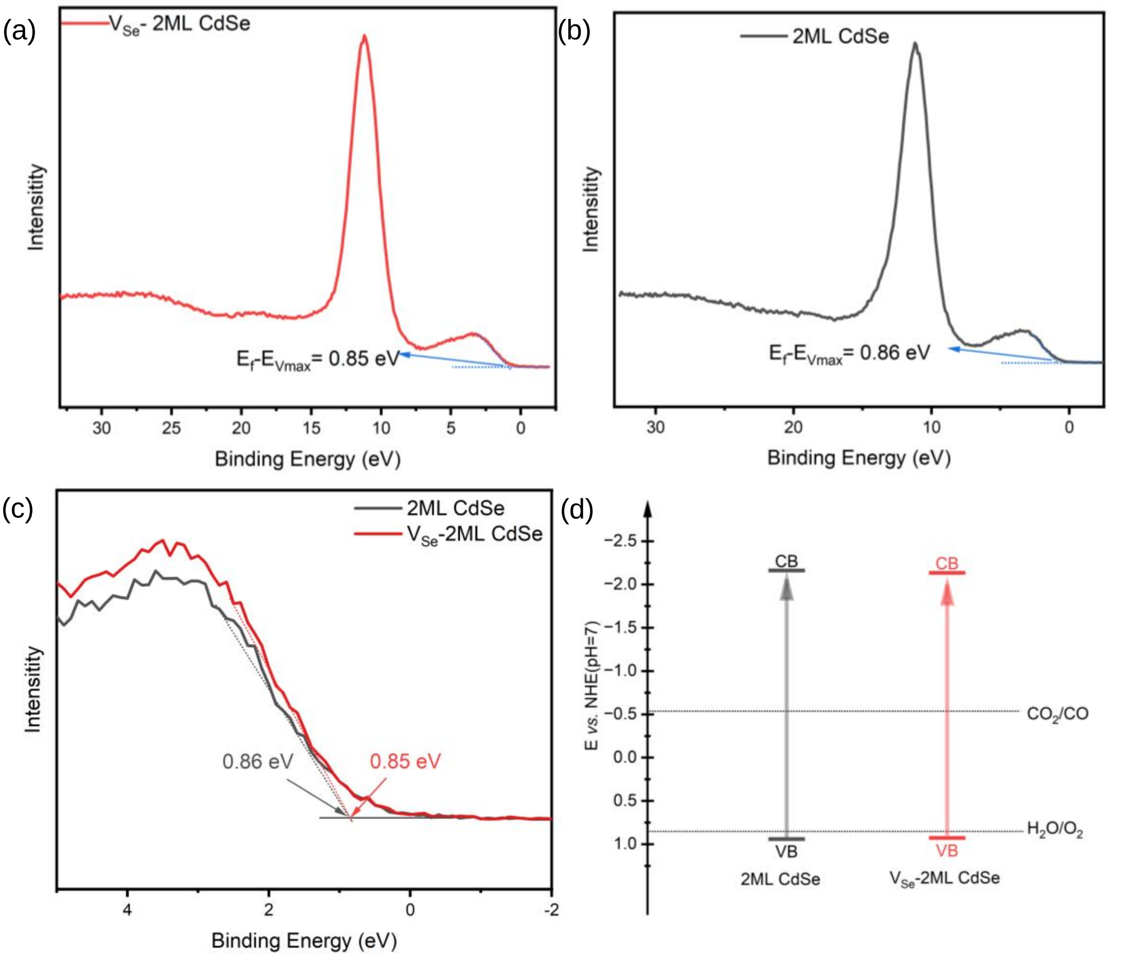

Figure S31. XPS valence band spectra of (a) V_{Se}-2ML CdSe, and (b) 2ML CdSe, (c) XPS valence band spectra of V_{Se}-2ML CdSe, and 2ML CdSe in high resolution scale of banding energy, and (d) energy band structure diagram of V_{Se}-2ML CdSe and 2ML CdSe. The E_{VB} of the corresponding standard hydrogen electrode (E_{VB}, NHE) can be calculated according to the following formula: $E_{VB,\ NHE} = \varphi + E_{VB,\ XPS} - 4.44$, where φ is the work function of the instrument (4.5 eV). Thus, the $E_{VB,\ NHE}$ of 2ML CdSe and V_{Se}-2ML CdSe is calculated to be 0.92 and 0.91 eV, respectively. Besides, the band gap relationship of different samples can be calculated according to the empirical formula: valence band potential (E_{VB}) = $E_{CB} + E$g.

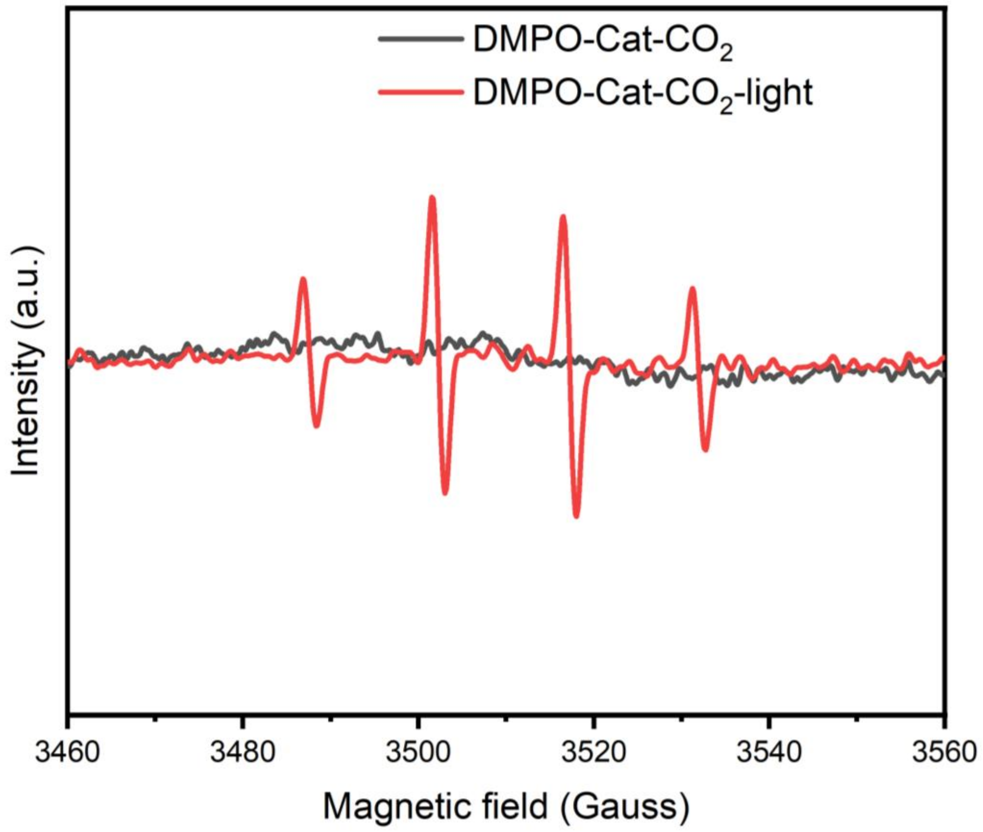

Figure 32. EPR signal for capturing hydroxyl radical (·OH).

As monitoring O_2 is essential in the gas-solid CO_2 photoreduction system involving water. Unfortunately, since we used nitrogen (N_2) as the carrier gas for gas chromatography (GC), direct and effective detection of O_2 through GC was not feasible. Therefore, we employed an indirect approach to demonstrate O_2 evolution during the reaction process.

We believe that O_2 evolution originated from the oxidation half-reaction involving water (H_2O) according to previous literature.[12] In this study, the reaction was a gas-solid phase process with only CO_2, H_2O, and the V_{Se}-2ML CdSe catalyst present in the reactor. Considering energy and mass conservation, apart from CO_2 participating in the reduction half-reaction, the only possible species involved in the oxidation half-reaction was either H_2O or the V_{Se}-2ML CdSe catalyst. However, through various characterizations, including EPR, UV-Vis, XRD, and TGA, we confirmed that V_{Se}-2ML CdSe remained stable during the reaction and served only as a catalyst. Thus, H_2O was responsible for the oxidation half-reaction. We further analyzed possible pathways for the oxidation half-reaction involving H_2O. One pathway was the direct formation of O_2 (eq (5)), while another involved the formation of hydroxyl radicals (·OH) (eq (6)).[13] Considering the bandgap structure of our sample (**Figure S31**), it could directly generate O_2 during the H_2O oxidation half-reaction but could not form hydroxyl radicals (·OH) directly. Therefore, we concluded that H_2O participates in the oxidation half-reaction, resulting in O_2 evolution.

Furthermore, a literature review indicated that our sample's band structure supported the reduction of O_2 to form H_2O_2 (eq (7)) or superoxide radicals ($^{\bullet}O_2^-$) (eq (8)). Since under light irradiation, H_2O_2 can directly decompose into hydroxyl radicals (·OH) (eq (9)),[14] we inferred that capturing either hydroxyl radicals (·OH) or superoxide radicals ($^{\bullet}O_2^-$) during the reaction would provide experimental evidence for O_2 evolution. To verify this, we conducted radical trapping experiments in a liquid-solid reaction system. The procedure was as follows: first, CO_2 was bubbled through H_2O until saturation, and any residual oxygen in the water was purged. Next, we added DMPO as a radical trapping agent and performed EPR measurements under both dark and illuminated conditions. Notably, we successfully detected the ·OH EPR signal under light irradiation (**Figure S32**). This provided further evidence supporting the involvement of H_2O in the oxidation half-reaction, leading to O_2 evolution during the reaction process.

$$2H_2O + 4h^+ \rightarrow O_2 + 4\,H^+ \quad (E^0 = +0.81\text{ V } vs.\text{ NHE}) \quad (5)$$

$$H_2O + h^+ \rightarrow \cdot OH + H^+ \quad (E^0 = +2.32\text{V } vs.\text{ NHE}) \quad (6)$$

$$O_2 + 2H^+ + 2e^- \rightarrow H_2O_2 \quad (E^0 = +0.68\text{ V } vs.\text{ NHE}) \quad (7)$$

$$O_2 + e^- \rightarrow {}^{\bullet}O_2^- \quad (E^\circ = -0.33\text{ V } vs.\text{ NHE}) \quad (8)$$

$$H_2O_2 \xrightarrow{hv} \cdot OH \quad (9)$$

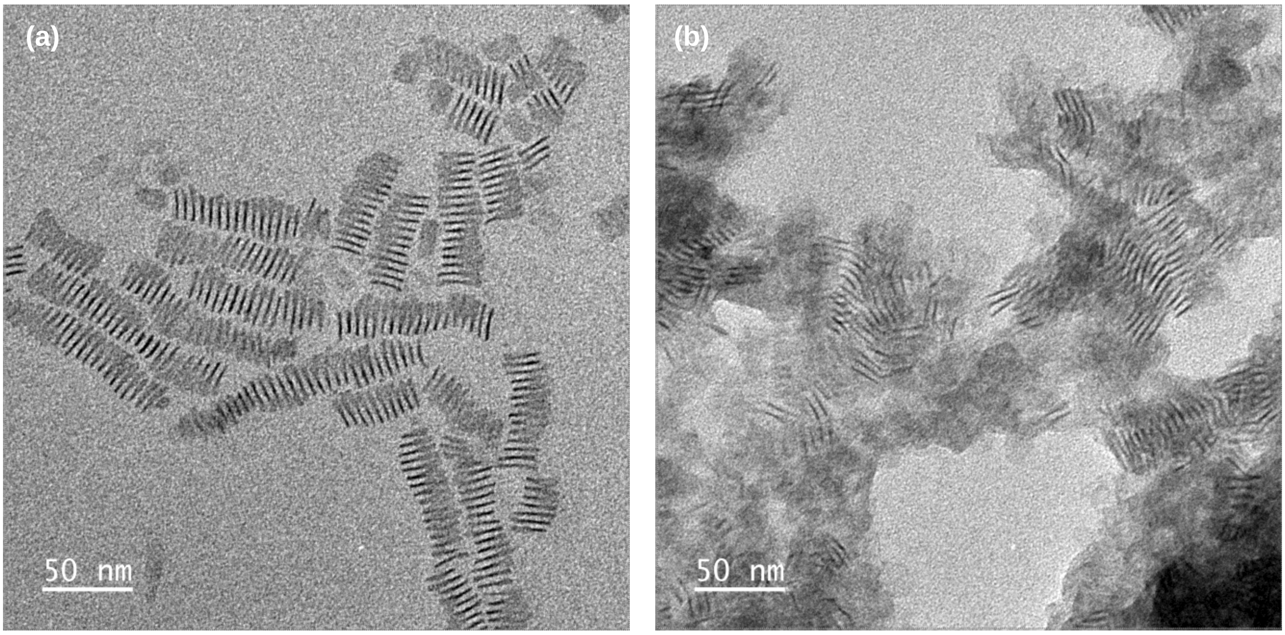

Figure S33. TEM images of (a) 4ML CdSe and (b) V_{Se}-4ML CdSe.

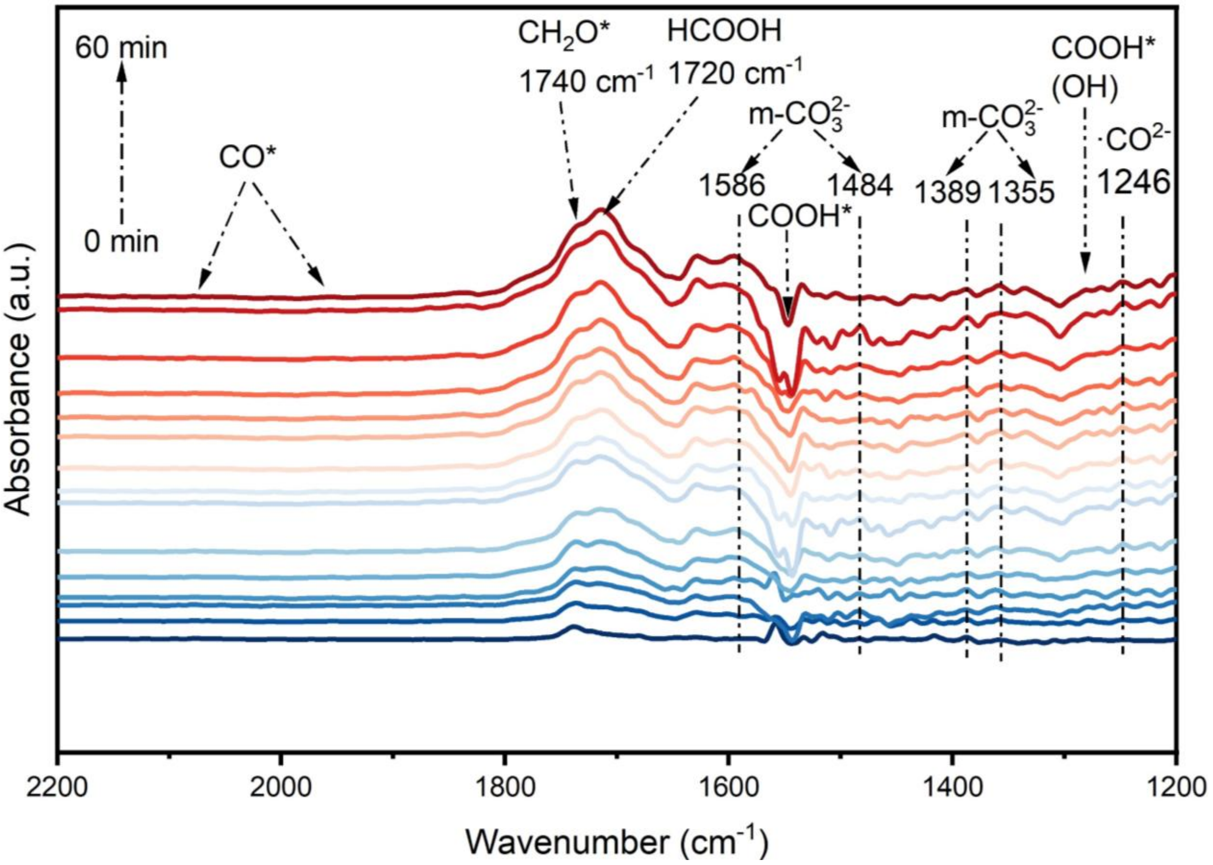

Figure S34. In-situ DRIFTS of 2ML CdSe in the presence of CO_2 and H_2O vapor within 60 min illumination.

Table S1. ICP data of different CdSe NPLs samples.

Samples	Mass concentration (mg L^{-1})<br>(Cd, Se, In)	Mole ratio<br>(Cd: Se: In)
2ML CdSe	3.128, 1.48, x[a]	1.50: 1: x
V_{Se}-2ML CdSe	2.272, 1.029, -0.0127	1.55: 1: x
2ML CdSe-In$(NO_3)_3$	2.104, 0.95, 0.0014	1.56: 1: 0.001
4ML CdSe	0.6014, 0.3373: x	1.25: 1: x
V_{Se}-4ML CdSe	0.6109, 0.3378: x	1.27: 1: x

[a] x denoted as no detectable

Table S2. Comparison of photocatalytic activity with reported CO_2 to CO conversion based on the gas-solid systems

No.	Ligut Source	Catalyst	Sacrificial agent	CO (μmol g^{-1} h^{-1})	Reference
1	300W Xe lamp, λ>420 nm	40%C-$In_2O_3/W_{18}O_{499}$	-	135.82	*Chinese J. Catal* 2023[15]
2	150-W Xe lamp, AM 1.5G filter	COF/TiO_2	-	91	*Adv. Sci.* 2023[16]
3	400 W Hg lamp, λ > 400 nm	RuRu′/ZrNBr-BK	-	4.83	*Angew. Chem.Int. Ed.* 2023[17]
4	300 W Xe lamp	PTPP-DA	TEOA	465	*ACS Sustainable Chem. Eng.* 2022[18]
5	300 W Xe lamp	BP/$Bi_{19}Br_3S_{27}$	-	395.7	*Appl. Catal. B Environ. Energy* 2022[19]
6	300 W Xe lamp, λ > 320 nm, 346.5 mWcm^{-2}	Au@ (PbS-CdS)	-	410	*Nano Energy* 2022[20]
7	300 W Xe lamp	(ROV)-DUC-$PbBiO_2Cl$		16.02	*Adv. Funct. Mater.*2022[21]
8	300 W Xe lamp	CdS/TiO_2:Cu	H_2S	781.3	*Adv. Funct. Mater.*2022[22]
9	Xe lamp with λ ≥ 420 nm	CdS@COF	-	600	*ACS Catal.* 2022[23]
10	300 W Xe lamp, λ>420 nm	CdSe/CdS	-	65.8	*Nat. Commun.* 2022[24]
11	300 W Xe lamp, λ > 400 nm, 100 mWcm^{-2}	ZnSe/$CsSnCl_3$	IPA	128.32	*ACS Nano* 2022[25]
12	300 W Xe lamp, λ = 320–780 nm	g-C_3N_4/TiN	TEOA	210.5	*Appl. Catal. B Environ. Energy* 2021[26]
13	300 W Xe lamp, λ > 400 nm	V_O-In_2O_3	TEOA	63.3	*Appl. Catal. B Environ. Energy* 2021[27]
14	300 W Xe lamp, λ>420 nm	FeOOH/CdS	-	12.55	*Angew.Chem.Int.Ed.* 2021[28]
15	300 W Xe lamp, 1 Sun	WP-NC/g-C_3N_4	-	376	*Appl. Catal. B Environ. Energy* 2021[29]
16	300 W Xe lamp, λ>420 nm	(g-C_3N_4)/CdSe-DETA)	-	25.87	*ACS Appl. Energy Mater.* 2021[30]
17	400 W Xe lamp	OD-ZnO/C	-	118.8	*J. Catal.* 2020[31]
18	300 W Xe lamp	2D/2D T-$SrTiO_3/CsPbBr_3$	-	120.2	*Chinese Chem Lett* 2023[32]
19	300 W Xe lamp, λ>420 nm	CPB-PCN	-	148.9	*Angew.Chem.Int.Ed.* 2018[33]
**20**	**300 W Xe lamp**	**V_{Se} CdSe NPL**	**-**	**2557.5**	**This work**

Supplementary References:

[1] A. Di Giacomo, C. Rodà, A. H. Khan, I. Moreels, *Chem. Mater.* **2020**, *32*, 9260.

[2] C. X. She, I. Fedin, D. S. Dolzhnikov, P. D. Dahlberg, G. S. Engel, R. D. Schaller, D. V. Talapin, *ACS Nano* **2015**, *9*, 9475.

[3] S. Shaw, J. L. Colaux, J. L. Hay, F. C. Peiris, L. Cademartiri, *Adv. Mater.* **2016**, *28*, 8900.

[4] S. Shaw, X. C. Tian, T. F. Silva, J. M. Bobbitt, F. Naab, C. L. Rodrigues, E. A. Smith, L. Cademartiri, *Chem. Mater.* **2018**, *30*, 5961.

[5] K. Li, Y. M. Cai, X. H. Yang, S. Wang, C. Teng, Y. Tian, Q. H. Min, W. L. Zhu, *Adv. Funct. Mater.* **2022**, *32*, 2113002.

[6] S. Sorcar, Y. Hwang, C. A. Grimes, S. In, *Mater. Today* **2017**, *20*, 507.

[7] G. Kressa., J. Furthmiiller, *Comp. Mater. Sci.* **1996**, *6*, 15.

[8] P. E. Blochl, *Phys. Rev. B Condens. Matter* **1994**, *50*, 17953.

[9] J. P. Perdew, J. A. Chevary, S. H. Vosko, K. A. Jackson, M. R. Pederson, D. J. Singh, C. Fiolhais, *Phys. Rev. B Condens. Matter* **1992**, *46*, 6671.

[10] N. C. Anderson, M. P. Hendricks, J. J. Choi, J. S. Owen, *J. Am. Chem. Soc.* **2013**, *135*, 18536.

[11] E. L. Rosen, R. Buonsanti, A. Llordes, A. M. Sawvel, D. J. Milliron, B. A. Helms, *Angew. Chem. Int. Ed.* **2012**, *51*, 684.

[12] X. He, Z. Gan, S. Fisenko, D. Wang, H, M. El-Kaderi, W.N. Wang, *ACS Appl. Mater. Interfaces* **2017,** 9, 9688.

[13] a) Z. Sun, N. Talreja, H. Tao, J. Texter, M. Muhler, J. Strunk, J. Chen, *Angew. Chem. Int. Ed.* 2018, 57, 7610; b) X. Zeng, Y. Liu, X. Hu, X. Zhang, *Green Chem.*, **2021**, *23*, 1466.

[14] S. Goldstein, D. Aschengrau, Y. Diamant, J. Rabani, *Environ. Sci. Technol.* **2007**, *41*, 7486.

[15] H. He, Z. Wang, K. Dai, S. Li, J. Zhang, *Chin. J. Catal.* **2023**, *48*, 267.

[16] A. Putta Rangappa, D. Praveen Kumar, K. H. Do, J. Wang, Y. Zhang, T. K. Kim, *Adv. Sci.* **2023**, *10*, e2300073.

[17] Y. Bao, S. Du, K. Shibata, X. Guo, Y. Kamakura, Z. Feng, Y. Huang, O. Ishitani, K. Maeda, F. Zhang, *Angew. Chem. Int. Ed.* **2023**, *62*, e202214273.

[18] X. Yu, S. Tian, F. Zhang, G. Gao, C. Zhang, Y. Han, S. Ji, H. Guo, X.-H. Jin, *ACS Sustainable Chem. Eng.* **2022**, *10*, 16182.

[19] R. Niu, Q. Liu, B. Huang, Z. Liu, W. Zhang, Z. Peng, Z. Wang, Y. Yang, Z. Gu, J. Li, *Appl. Catal., B* **2022**, *317*, 121727.

[20] X. Wan, Y. Gao, M. Eshete, M. Hu, R. Pan, H. Wang, L. Liu, J. Liu, J. Jiang, S. Brovelli, J. Zhang, *Nano Energy* **2022**, *98*, 107217.

[21] B. Wang, W. Zhang, G. Liu, H. Chen, Y. X. Weng, H. Li, P. K. Chu, J. Xia, *Adv. Funct. Mater.* **2022**, *32*, 2202885.

[22] K. Li, Y. Cai, X. Yang, S. Wang, C. Teng, Y. Tian, Q. Min, W. Zhu, *Adv. Funct. Mater.* **2022**, *32*, 2113002.

[23] L. Zou, R. Sa, H. Zhong, H. Lv, X. Wang, R. Wang, *ACS Catal.* **2022**, *12*, 3550.

[24] H. Li, C. Cheng, Z. Yang, J. Wei, *Nat. Commun.* **2022**, *13*, 6466.

[25] N. Li, X. Chen, J. Wang, X. Liang, L. Ma, X. Jing, D. L. Chen, Z. Li, *ACS Nano* **2022**, *16*, 3332.

[26] Q. Zhu, Y. Xuan, K. Zhang, K. Chang, *Appl. Catal., B* **2021**, *297*, 120440.

[27] L. Li, C. Guo, J. Ning, Y. Zhong, D. Chen, Y. Hu, *Appl. Catal., B* **2021**, *293*, 120203.

[28] J. Bian, Z. Zhang, J. Feng, M. Thangamuthu, F. Yang, L. Sun, Z. Li, Y. Qu, D. Tang, Z. Lin, F. Bai, J. Tang, L. Jing, *Angew. Chem. Int. Ed.* **2021**, *60*, 20906.

[29] X. Zhang, J. Yan, F. Zheng, J. Zhao, L. Y. S. Lee, *Appl. Catal., B* **2021**, *286*, 119879.

[30] Y. Huo, J. Zhang, K. Dai, C. Liang, *ACS Appl. Energy Mater.* **2021**, *4*, 956.

[31] L.-Y. Lin, C. Liu, T.-T. Hsieh, *J. Catal.* **2020**, *391*, 298.

[32] S.-X. Yuan, K. Su, Y.-X. Feng, M. Zhang, T.-B. Lu, *Chin. Chem. Lett.* **2023**, *34*, 107682.

[33] M. Ou, W. Tu, S. Yin, W. Xing, S. Wu, H. Wang, S. Wan, Q. Zhong, R. Xu, *Angew. Chem. Int. Ed.* **2018**, *57*, 13570.
